# Performance of a GPU- and time-efficient pseudo-3D network for magnetic resonance image super-resolution and motion artifact reduction

**DOI:** 10.1038/s41598-026-43804-1

**Published:** 2026-03-21

**Authors:** Hao Li, Jianan Liu, Marianne Schell, Tao Huang, Arne Lauer, Katharina Schregel, Jessica Jesser, Dominik F. Vollherbst, Martin Bendszus, Sabine Heiland, Tim Hilgenfeld

**Affiliations:** 1 Department of Neuroradiology, Heidelberg University Hospital, Im Neuenheimer Feld 400, 69120 Heidelberg, Germany; 2 Momoni AI, Gothenburg, Sweden; 3 College of Science and Engineering, James Cook University, Smithfield, Australia; 4 Department of Radiology, Section Neuroradiology, Jena University Hospital, Jena, Germany

**Keywords:** Magnetic resonance imaging, 3D super-resolution reconstruction, 3D motion artifact reduction, Uncertainty for image quality estimation, GPU and time efficiency, Computational biology and bioinformatics, Engineering, Health care, Mathematics and computing, Medical research

## Abstract

Minimizing acquisition time and motion-artifacts remains challenging in magnetic resonance imaging (MRI) with demands on high-resolution images for accurate diagnosis and treatment. Deep learning-based image restoration offers promising solution by generating high-resolution and artifact-free MR images from low-resolution or motion-corrupted data. To facilitate practical deployment in clinical workflows, this study presents a time-/GPU-efficient framework using 2D network (TS-RCAN) for pseudo-3D MRI super-resolution reconstruction (SRR) and motion-artifact reduction (MAR). Optimal down-sampling factors were identified to balance SRR accuracy and acquisition time. MAR training used a standardized method to induce controllable motion-artifacts of varying severity. Network performance was benchmarked against state-of-the-art 3D networks. Results showed the down-sampling factor $1\times 1\times 2$ for $\times 2$ acceleration and $2\times 2\times 2$ for $\times 4$ acceleration achieved optimal SRR performance. TS-RCAN outperformed most 3D networks by > 0.01/1.5 dB in SSIM/PSNR while reducing GPU load and inference time by up to 90%. For MAR, TS-RCAN exceeded UNet by up to 0.014/1.48 dB in SSIM/PSNR. Additionally, uncertainty estimation correlated with image quality metrics, enabling accuracy prediction without ground truth. TS-RCAN provides an efficient, accurate framework for SRR and MAR with practical relevance to clinical MRI, and offers a flexible basis for future extension to other imaging contrasts and pathological cases.

**Supplementary Information:**

The online version contains supplementary material available at 10.1038/s41598-026-43804-1.

## Introduction

Magnetic resonance imaging (MRI) is widely used in diverse medical applications to support accurate diagnosis. However, its potential is often constrained by spatial resolution and acquisition time. High-resolution magnetic resonance imaging provides more details but requires longer scans, which also increases the likelihood of motion artifacts that degrade image quality. Recent advances in deep learning offer promising solutions for improving image quality through super-resolution reconstruction (SRR) and motion artifact reduction (MAR).

Parallel imaging (PI) is widely used in clinical MRI to reduce scan time, but in practice the achievable acceleration is typically limited to moderate factors (approximately 2–3) due to SNR loss and g-factor-related noise amplification. SRR represents a different strategy for addressing the acquisition-resolution trade-off by reconstructing high-resolution (HR) images from lower-resolution (LR) data. As such, SRR can be applied either as a standalone approach or in parallel with PI, offering an additional degree of freedom to reduce acquisition time or to make very high-resolution protocols more feasible for clinical use.

SRR has progressed markedly with deep learning techniques. This data-driven approach trains networks on HR and LR image pairs to extract pixel-level features and generate super-resolution (SR) images. Dong et al.^1,2^ introduced a 2D convolutional neural network (CNN) for SRR. While most SRR studies in radiology employ 2D networks^3,4^, both computed tomography (CT) and MRI capture inherently 3D structures. Processing slices independently can result in misalignment between adjacent slices. To address this, 3D CNNs are preferred for modeling 3D spatial features^5^ and have shown superior performance in MRI SRR^6–9^. Yet, their computational demands are much higher, requiring substantial GPU resources and longer inference times, limiting clinical use. Although Chen et al.^6,7^ proposed more efficient 3D networks, the gap in GPU usage and inference time between 2 and 3D CNNs remains large. Recent studies using 2D networks for 3D SR typically adopt multi-network strategies, processing slices along different orientations with separate networks before integration^10–12^. While effective, these approaches increase model complexity and training cost.

Down-sampling factors also critically affect reconstruction complexity, acquisition time, and overall accuracy. In 2D network studies, factors such as $2\times 2\times 1$ (frequency-encoding(FE)$\times$phase-encoding(PE)$\times$slice-encoding(SL)) and $4\times 4\times 1$ are commonly used^13,14^. For 3D networks, down-sampling strategies, particularly with through-plane down-sampling, such as $1\times 1\times 2$, $1\times 1\times 4$ or $2\times 2\times 2$ are often applied^6,15–17^. These factors directly influence acquisition acceleration and reconstruction difficulty. A systematic analysis of down-sampling factors is thus essential to optimize GPU use, accelerate acquisition, and preserve SRR accuracy, yet this gap remains unaddressed.

Motion artifacts (MA) are another common challenge in clinical MRI^18^, often compromising diagnostic accuracy^19,20^. Deep learning methods have shown promise in MAR^21–27^. Although early work used a 3D U-Net for MAR^21^, most subsequent studies employed 2D U-Net architectures^22–26^. This shift is largely driven by practical considerations, as 3D CNNs incur substantially higher memory and computational costs, making them harder to train and deploy, whereas 2D models offer greater efficiency and robustness in practice. However, purely 2D approaches ignore through-plane correlations, which can lead to inconsistent artifact correction across slices. Similar to the SRR task, these limitations motivate our use of a thin-slab pseudo-3D design that preserves computational efficiency while capturing partial through-plane information for improved MAR performance.

Another critical concern is the accuracy of the reconstructed images, since anatomical misrepresentations can bias diagnosis and treatment decisions. To address this, Tanno et al.^28^ and Qin et al.^29^ introduced uncertainty prediction^30^. However, these approaches do not distinguish between noise-driven aleatoric uncertainty and model-driven epistemic uncertainty due to out-of-distribution (OOD) data^31,32^. Aleatoric uncertainty is inherent and cannot be eliminated, while epistemic uncertainty reflects knowledge gaps and can be reduced with broader training data. Since CNN performance in clinical settings often deviates from training conditions where ground truth (GT) images are unavailable, a reliable method to estimate the accuracy of reconstructed MR images is urgently needed.

This study hypothesizes that a pseudo-3D framework using single 2D CNN can provide a unified, GPU-efficient solution for 3D SRR and MAR, achieving performance comparable to or exceeding that of specialized 3D CNNs. Additionally, we propose a pixel-wise uncertainty estimation method to assess image accuracy in the absence of GT images.

## Methods and materials

### MR image restoration Network

We adapted the 2D residual channel attention network (RCAN) architecture^33^ into thin-slab RCAN (TS-RCAN), enabling efficient end-to-end pseudo-3D SRR and MAR while remaining computationally feasible on standard GPUs. RCAN employs channel-attention mechanisms to adaptively reweight feature channels, which is particularly well suited to our thin-slab formulation, where adjacent slices along the through-plane dimension are mapped to the channel dimension to capture inter-slice contextual information. The network structure consists of residual groups (RG) built from residual channel attention blocks (RCAB), followed by an up-sampling module for in-plane scaling when required. The network processes low-resolution (LR) inputs (or MA images), either a single slice or a thin slab of multiple slices, to generate the corresponding 2D or 3D images. The network structure can be found in Supplementary Fig. S1.

The multichannel nature of the 2D network allows direct handling of 3D data through the channel dimension. In this approach, both input and output use multiple channels, mapping the third dimension of a 3D patch to the channel dimension. For input with $M$ ($M\le 5$ in our experiments) and patch size $M\times H\times W$ ($M$: number of channels, $H$: height of the matrix, $W$: width of the matrix), the first convolutional layer has a kernel size of $B\times M \times H\times W$ ($B$: batch size). This setup acts like a 3D kernel of size $B\times 1\times M \times H\times W$ without padding. The network extracts and compresses multi-slice features into a multi-channel feature map using various filters, which hidden layers then use to reconstruct high-quality images. The final convolution outputs the expected slice count of the target patch. Compared with conventional 3D CNNs, a thinner slab input was adopted since 2D kernels do not stride along the channel dimension.

Furthermore, traditional network-based ensembles^34^ and data-based ensembles^35^ increase training time and operational complexity. To address this, we used a simple and effective self-ensemble strategy. Thin-slab input patches ensured each slice appeared in different positions across patches, allowing the same slice to be processed along multiple paths and yielding diverse outputs.

### Down-sampling factors for super-resolution

To generate synthetic LR images, we employed k-space truncation, a widely used method for simulating real LR MRI acquisitions^14^. Therefore, we also adopted this method to generate synthetic LR images in our study.

**Fig. 1:**
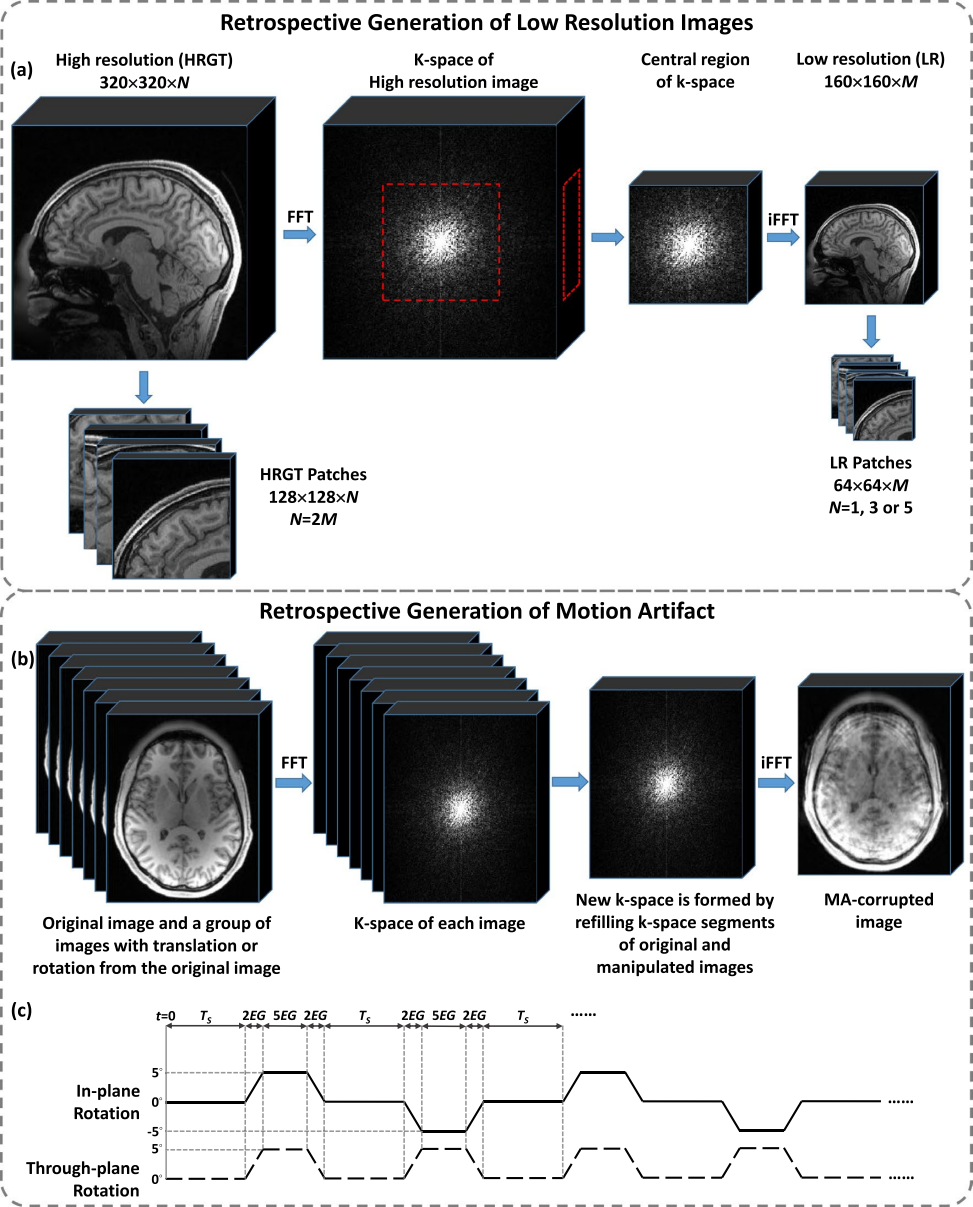
Retrospective generation of 3D low resolution and motion artifact-corrupted MR images. (**a**) 3D low resolution images generation with a scale factor of $2\times 2\times 2$ and patch cropping. (**b**) Motion artifact (MA) generation in MR images. (**c**) Motion pattern for MA generation.

HR images were transformed into k-space using a 3D Fast Fourier Transform (FFT), truncated in three dimensions according to selected scale factors to retain only the central region, and then converted back into LR images via a 3D inverse FFT (iFFT). Finally, voxel intensities of HR and LR images were rescaled to [0,1]. The 3D LR image generation process is illustrated in Fig. 1a.

The acquisition time for 3D MRI mainly depends on the number of phase-encoding (PE) and slice-encoding (SL) steps. Thus, down-sampling in these directions effectively reduces scan time. All acquisition parameters, particularly the imaging volume (field of view), were assumed identical. Only the matrix size was reduced along different directions. Because the imaging volume remained fixed, reducing the matrix size led to a proportional increase in voxel size along the corresponding direction and a proportional reduction in acquired k-space lines. Under these controlled conditions, the reduction in k-space lines directly results in a proportional reduction in acquisition time. Therefore, in this study, the acceleration factor corresponds directly to the proportional reduction in scan time and is determined by the product of the sub-sampling ratios along the PE and SL directions.

Often in MRI sequences, PE and frequency-encoding (FE) are typically adjusted simultaneously to preserve perfect or relative symmetric in-plane resolution. Therefore, symmetric in-plane sub-sampling was adopted in this study to ensure best compatibility with common clinical protocols. This, however, unnecessarily discards data in FE direction and complicates SR reconstruction. Slice thickness is independent of in-plane resolution and provides more flexible down-sampling. Here, the three down-sampling factors are ordered as frequency-encoding (FE) × phase-encoding (PE) × slice-encoding (SL), and the term “acceleration” is used to denote the effective reduction ratio in sampled k-space lines that lead to actual scan-time reduction. For instance, $\times 2$ acceleration can be achieved with $2\times 2\times 1$, discarding 75% of the K-space, or $1\times 1\times 2$, discarding only 50%.

The complexity of image reconstruction depends not only on the amount of discarded data but also on the loss of low-frequency components, which are critical for SRR. We therefore examined multiple down-sampling factors and their impact on SRR: $2\times 2\times 1$ and $1\times 1\times 2$ for $\times 2$ acceleration, and $4\times 4\times 1$, $2\times 2\times 2$, and $1\times 1\times 4$ for $\times 4$ acceleration, following standard MRI protocols. These systematic variations of down-sampling factors and input slice numbers served both as performance evaluation and ablation studies to isolate their effects.

After down-sampling, HR and LR images were cropped into patches in sagittal plane to reduce computational load. HR images were divided into $128\times 128$ patches with 32-voxel overlap, while LR images were cropped into $64\times 64$ patches with 16-voxel overlap for scale factor 2, and $32\times 32$ patches with 8-voxel overlap for scale factor 4. Each LR patch contained 1, 3, or 5 consecutive slices, with $n-1$ overlapping slices between neighbors, while HR patches contained slice number equal to 1, 3, or 5 times the through-plane scale factor. For comparative 3D networks, LR images were interpolated to the HR matrix size^6–8,36^, and both HR and LR were cropped into $64\times 64\times 64$ patches with 32-voxel overlap. During the inference, in-plane patches are combined by discarding border regions and directly tiling the remaining central areas. Along the through-plane direction, outputs from overlapping thin slabs are first stitched slice-wise to form multiple volumes, and then self-ensemble was performed by voxel-wise averaging the multiple volumes to obtain the final 3D reconstruction.

### Motion pattern and motion artifact quantification

A method involving the splicing of lines from multiple k-spaces was employed to simulate realistic MA in MR images. As shown in Fig. 1b, a series of images were generated by rotating the original volume in specific directions at defined angles. Both original and rotated images were transformed to k-space using FFT, and segments of the original k-space were replaced by those from the rotated images following a predefined pattern. This process is the most commonly used motion artifact simulation algorithm in MAR studies. To reduce computational resource requirements, axial-plane images were used for motion artifact correction, highlighting the potential of the network to process images in various orientations (e.g. sagittal and axial). No in-plane cropping was applied, and only through-plane cropping was performed, resulting in MA-corrupted inputs and corresponding ground-truth (GT) images with an in-plane size of $320\times 256$. Self-ensemble was also applied.

Previous studies often employed random movements to generate MA^25–27^, making artifact severity difficult to control or reproduce. To address this, we used simplified periodic motion patterns: 5-degree head rotations for in-plane motion and 5-degree head nodding for through-plane motion. Motion severity was regulated by adjusting duration and frequency, as illustrated in Fig. 1c. The minimal time unit was an echo group ($EG$), representing acquisition of consecutive echoes (analogous to TR in turbo spin-echo). All motion durations were integer multiples of EG. The complete pattern is defined as below:

1. At $t=0$, the patient stayed in the original position and stayed for ${T}_{S}$;
2. from $t={T}_{S}$ to $t={T}_{S}+2EG$, the patient’s head rotated to the left for 5 degrees;
3. from $t={T}_{S}+2EG$ to $t={T}_{S}+7EG$, the patient’s head stayed at the position of 5 degrees to the left;
4. from $t={T}_{S}+7EG$ to $t={T}_{S}+9EG$, the patient’s head rotated back to the starting position;
5. from $t={T}_{S}+9EG$ to $t=2{T}_{S}+9EG$, the patient’s head stayed in the starting position;
6. from $t=2{T}_{S}+9EG$ to $t=2{T}_{S}+18EG$, the patient’s head rotated to the right and returned to the starting position following the same process of steps 2 to 4;
7. from $t=2{T}_{S}+18EG$ to $t=3{T}_{S}+18EG$, the patient’s head stayed in the starting position.

Steps 2 to 7 were repeated until the entire k-space was filled, and the severity of MA was adjusted via ${T}_{s}$ and $EG$.

In this study, ${T}_{s}$ was set to $9EG, 18EG, 36EG$ and $72EG$, resulting in k-space corruption ratios of 50%, 33%, 20% and 11%, respectively. Each $EG$ contained 80 echoes, and a centric trajectory was selected for k-space filling. As a result, the image quality metrics for different MA severity levels followed a linear trend.

### Uncertainty

Previous studies have shown that pixel-wise aleatoric uncertainty can be estimated by incorporating Gaussian negative log-likelihood (NLL) loss into neural networks^37^, and this has been applied to MRI SRR^28,29^. Aleatoric uncertainty reflects inherent noise in training data, which can be reduced by enlarging datasets and is therefore not the main concern in clinical image restoration. The more critical challenge arises from out-of-distribution (OOD) data, where images from different patients or scanners may vary even under identical protocols. This is captured by epistemic uncertainty^30^, making it essential for robust medical image reconstruction.

In this study, both pixel-wise aleatoric and epistemic uncertainties were estimated using evidential regression^32^. Evidential deep learning treats training as evidence acquisition, with each sample contributing to a higher-order evidential distribution. Sampling from these yields lower-order likelihood functions. Unlike Bayesian networks that place priors on weights, evidential learning places priors on the likelihood itself. By training the network to output evidential hyperparameters, both aleatoric and epistemic uncertainties can be estimated without sampling. Amini et al.^32^ proposed estimating a posterior distribution $q\left(\mu ,{\sigma }^{2}\right)$ approximating a Normal Inverse − Gamma (NIG) distribution $p\left(\mu ,{\sigma }^{2}|\gamma ,v,\alpha ,\beta \right)$ , serving as the Gaussian conjugate prior. Predictions and uncertainty estimations are calculated as follows:

1 $$\begin{array}{c}Prediction:E\left[\mu \right]=\gamma \end{array}$$

2 $$\begin{array}{c}Aleatoric:E\left[\sigma 2\right]=\frac{\beta }{\alpha -1}\end{array}$$

3 $$\begin{array}{c}Epistemic:Var\left[\mu \right]=\frac{\beta }{v\left(\alpha -1\right)}\end{array}$$

We further analyzed correlations between epistemic uncertainty and image quality metrics, structural similarity index (SSIM)^38^ and peak signal-to-noise ratio (PSNR). Linear and exponential regressions were applied to test datasets, and the resulting curves were used to estimate SSIM and PSNR for reconstructed images when GT was unavailable.

### Loss functions

Previous studies have employed various types of loss functions, each designed to refine specific image features. In this study, we adopted the pixel-wise Charbonnier loss^39^, a differentiable approximation of the L1 loss, to prevent excessive smoothing effects during training^40^:

4 $$\begin{array}{c}{L}_{Char}=\frac{1}{N}{\sum }_{i=1}^{N}\sqrt{{\left(H{R}_{i}-S{R}_{i}\right)}^{2}+\epsilon }\end{array}$$

where $\epsilon$ is assigned as ${10}^{-4}$.

In addition, SSIM loss has become increasingly popular for encouraging networks to reconstruct images with high structural similarity to GT^14^. In this study, we enhanced the importance of SSIM by applying the square of the SSIM value within the L1 loss:

5 $$\begin{array}{c}{L}_{SSIM}=\frac{1}{N}{\sum }_{i=1}^{N}1-SSIM{\left(S{R}_{i},H{R}_{i}\right)}^{2}\end{array}$$

For image restoration, we combined both the Charbonnier loss and the SSIM loss as a weighted sum:

6 $$\begin{array}{c}Loss={L}_{Char}+{w}_{1}{L}_{SSIM}\end{array}$$

where ${w}_{1}=0.5$ was chosen to provide strong quantitative performance and stable training behavior in our experiments.

Additionally, when generating uncertainty maps, we employed the Normal-Inverse-Gamma (NIG) loss^32^:

7 $$\begin{array}{c}{L}_{NIG}={L}_{NLL}+\lambda {L}_{Reg}\end{array}$$

8 $$\begin{aligned} {\mathrm{with}}\quad L_{NLL} & = \frac{1}{2}log\left( {\frac{\pi }{v}} \right) - \alpha log\left( \Omega \right) + \left( {\alpha + \frac{1}{2}} \right)log\left( {\left( {y - \gamma } \right)^{2} v + \Omega } \right) \\ & \quad + log\left( {\frac{\Gamma \left( \alpha \right)}{{\Gamma \left( {\alpha + \frac{1}{2}} \right)}}} \right) \\ \end{aligned}$$

9 $$\begin{array}{c}\Omega =2\beta \left(1+v\right)\end{array}$$

10 $$L_{Reg} = \mid y - \gamma \mid \left( {2v + \alpha } \right)$$

$\alpha , \beta , \gamma$ and $v$ were the outputs of the network. $y$ was the GT.

In summary, our approach combined the weighted Charbonnier loss and SSIM loss for SRR and MAR, and NIG loss for evidential regression:

11 $$\begin{array}{c}Loss={L}_{Char}+{w}_{1}{L}_{SSIM}+{w}_{2}{L}_{NIG}\end{array}$$

with ${w}_{1}=0.5$ and ${w}_{2}=1$ for optimal performance in our study.

### Datasets

This study used T1-weighted (T1w) images from the Human Connectome Project (HCP) dataset^41^, which provides multi-contrast MRI scans from 1113 patients. The T1w images were acquired in the sagittal plane with a 3D MPRAGE sequence with $2\times$ parallel imaging on Siemens 3 T customized Skyra scanners with 32-channel head coil. The matrix size was $320\times 320\times 256$ with 0.7 mm isotropic resolution. For experiments, 80 patients were randomly chosen for training, 10 for validation, and 10 for testing, with no overlap among groups. To further validate the quantified correlation between uncertainty and image quality metrics, an additional 40 patients from the HCP dataset were used as an independent accuracy prediction set, separated from training, validation, and test groups.

For testing the generalization capacity, 10 independent patients were randomly selected from MR-ART dataset^42^. The MR-ART dataset contains T1-weighted images from 148 healthy volunteers. For each volunteer, three scans are available: one standard scan acquired without intentional motion, and two motion-corrupted scans, referred to as Motion 1 and Motion 2, in which the volunteer performed 5 and 10 head motion events during acquisition, corresponding to low- and high-severity motion artifacts, respectively. All images were acquired in sagittal plane with 3D MPRAGE sequence with $2\times$ parallel imaging on Siemens 3 T Prisma scanner with 20-channel head coil. The matrix size was $256\times 256\times 192$ with 1.0 mm isotropic resolution. Any data from MR-ART dataset was not used in retraining or fine-tuning the network.

### Implementation details

For TS-RCAN implementation, the architecture comprised 5 residual groups (RGs), each with 5 residual channel attention blocks (RCABs). Convolutional layers in shallow feature extraction used 64 filters. Training was conducted on a workstation with a Nvidia Quadro A6000 GPU using PyTorch 1.9. Each batch randomly extracted eight LR patches as inputs. The network was trained for 50 epochs with the ADAM optimizer (${\beta }_{1}=0.9,{\beta }_{2}=0.999$, and $\epsilon ={10}^{-8}$ ) and a cosine-decay learning rate from ${10}^{-4}$ to ${10}^{-8}$. Reconstructed image quality was evaluated with PSNR and SSIM. The Shapiro–Wilk test was employed to check the normal distribution of the data. For statistical analysis, one-way ANOVA with post hoc Tukey was applied to normally distributed data, and Kruskal–Wallis with post hoc Dunn’s test to non-normally distributed data. Mann–Whitney U test was used for head-to-head comparisons with state-of-the-art methods. Data processing was performed in MATLAB, and statistical analysis in GraphPad Prism.

## Experiments and results

### Dependency of SRR network performance on down-sampling factors

**Fig. 2:**
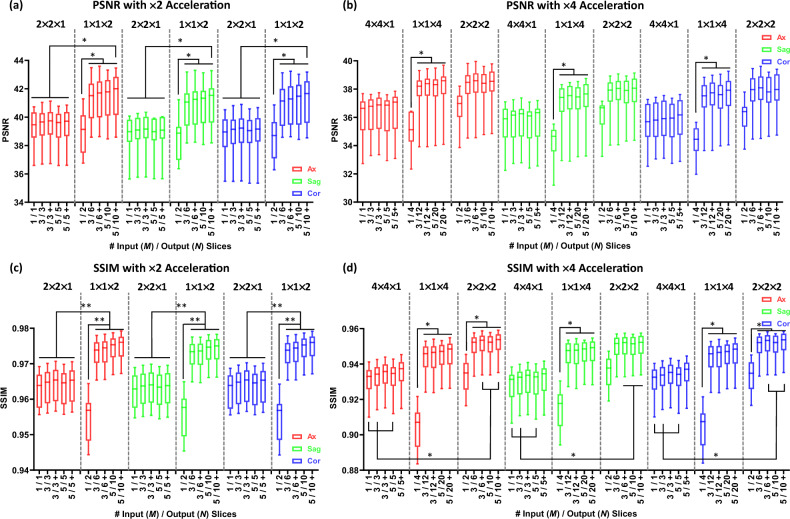
Dependency of SRR performance on down-sampling factors. The scale factors are grouped according to the acceleration factor. This figure summarizes SRR performance under different down-sampling factors and input/output slice settings, grouped by the overall acceleration factor. The horizontal axis denotes the number of input/output slices (M/N), indicating how many adjacent slices are jointly processed by the network. The down-sampling factors shown above each group (e.g., $1\times 1\times 2$) follow the *FE* × *PE* × *SL* convention defined in the Methods and are grouped by identical acceleration factors to allow direct comparison of different sampling strategies at the same acceleration level. Colors indicate the slice orientation of the reconstructed 3D volume (axial, sagittal, and coronal), where sagittal corresponds to the in-plane direction and axial/coronal correspond to through-plane directions. $1\times 1\times 2$ and $2\times 2\times 2$ down-sampling with $M\ge 3$ achieved the highest mean values of SSIM/PSNR values for $\times 2$ and $\times 4$ acceleration, respectively. For $\times 2$ acceleration, $1\times 1\times 2$ down-sampling with $M=5$ and self-ensemble significantly outperformed all cases of $2\times 2\times 1$ in PSNR and SSIM. For $\times 4$ acceleration, $2\times 2\times 2$ down-sampling with $M\ge 3$ and self-ensemble significantly outperformed $4\times 4\times 1$ in SSIM. (**a**)/(**c**) PSNR and SSIM of SR images with $\times 2$ acceleration ($2\times 2\times 1$ and $1\times 1\times 2$); (**b**)/(**d**) PSNR and SSIM of SR images with $\times 4$ acceleration ($4\times 4\times 1$, $1\times 1\times 4$ and $2\times 2\times 2$). ‘ + ’ represents the SRR with self-ensemble. * p < *0.05* and ** p < *0.0005*.

The mean SSIM and PSNR for the SRR of the factors using only in-plane down-sampling ($2\times 2\times 1$ and $4\times 4\times 1$) with a 3-slice input ($M=3$) outperformed those with $M=1$ by up to 0.004 / 0.3 dB, although the differences were not statistically significant (*p* values > 0.99). After reaching their peaks at $M=3$, both SSIM and PSNR slightly declined with 5-slice input ($M=5$), as shown in Fig. 2a,c. For through-plane down-sampling ($1\times 1\times 2$, $1\times 1\times 4$ and $2\times 2\times 2$), the mean SSIM and PSNR of SR images reconstructed with $M=3$ increased significantly compared to those with $M=1$ with significant difference in most of the cases (*p* values range: < 0.0001 to 0.03). With $M=5$, SSIM and PSNR values improved further, although a significant difference was not detected (*p* values > 0.99). Furthermore, the application of self-ensemble techniques improved the performance of the 3D SRR on all down-sampling factors.

Comparisons of SRR performance across different down-sampling factors with the same acceleration rates showed that for $\times 2$ acceleration, the mean PSNR value of $1\times 1\times 2$ down-sampling with $M=5$ and self-ensemble were significantly higher by more than 2.2 dB compared to $2\times 2\times 1$ with various configurations (*p* value range: 0.01 to 0.04). The mean SSIM values of $1\times 1\times 2$ down-sampling with multi-slice input ($M=3\, and\, 5$) were also significantly higher by more than 0.01 compared to $2\times 2\times 1$ with different input configurations ($M=1, 3 \,and \,5$) in all directions (*p* value range: < 0.0001 to 0.01).

For $\times 4$ acceleration, the $2\times 2\times 2$ down-sampling achieved the highest mean SSIM/PSNR values, exceeding those of $1\times 1\times 4$ by more than 0.003/0.3 dB and $4\times 4\times 1$ by over 0.02/1.9 dB, with statistically significant differences observed in certain cases (*p* value range: 0.01 to 0.05) as shown in Fig. 2b,d.

**Fig. 3:**
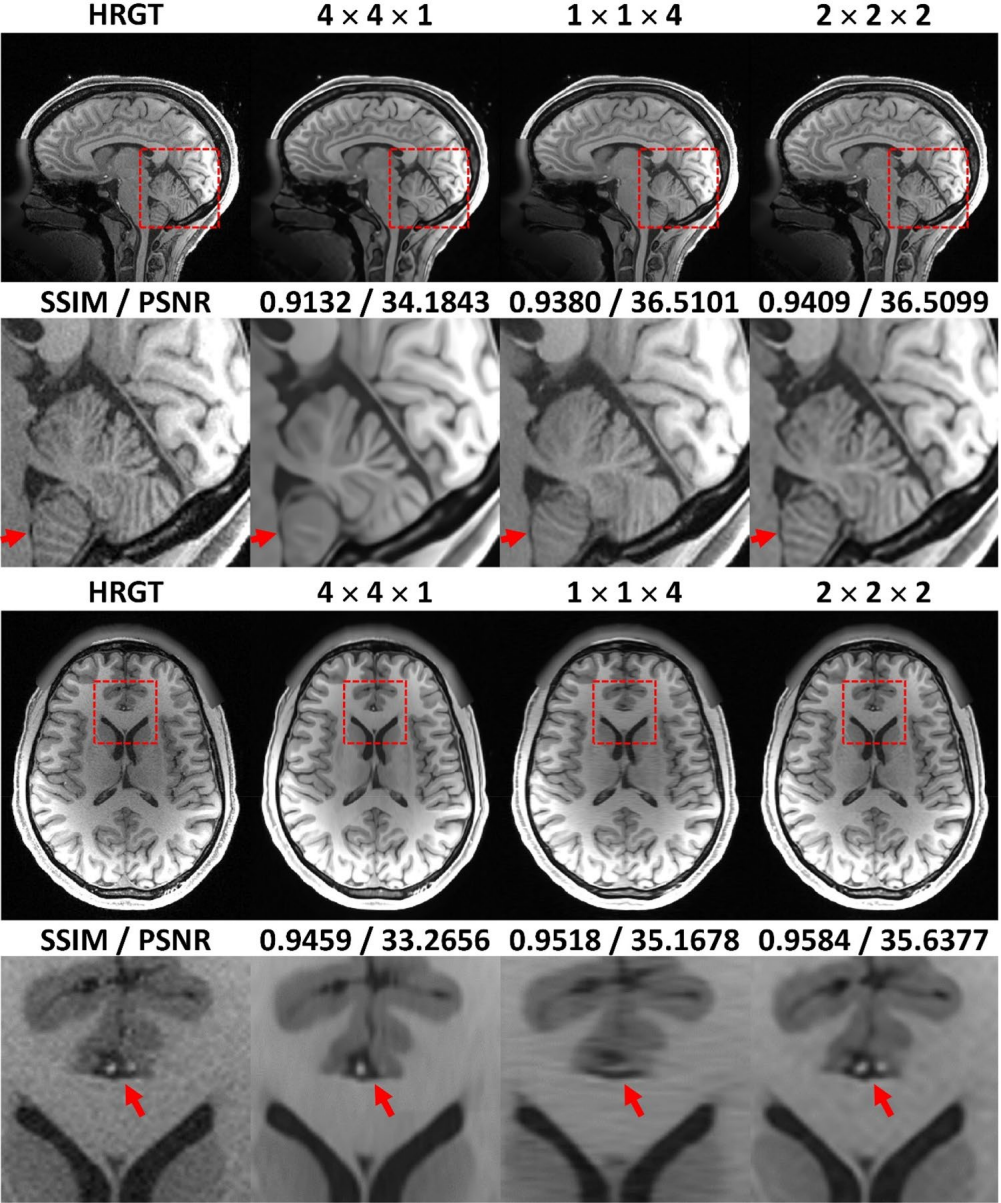
Qualitative comparison of 3D SRR with different down-sampling factors of $\times 4$ acceleration. Note the loss of anatomical details in the cerebellar grey-white-matter differentiability in the sagittal image (arrow) of $4\times 4\times 1$ down-sampling strategy, as well as the loss of one/both anterior cerebral arteries (arrows) in the axial image of the $4\times 4\times 1$ / $1\times 1\times 4$ down-sampling factors. Best qualitative results were achieved using the $2\times 2\times 2$ down-sampling factor.

The superior SSIM/PSNR values of $2\times 2\times 2$ down-sampling also led to more accurate representations of fine anatomical details (Fig. 3 and Supplementary Fig. S2). The reconstructed image from 4 × 4 × 1 lost numerous small anatomical structures in the sagittal plane, while 1 × 1 × 4 showed similar losses in the axial plane. In contrast, the image reconstructed from 2 × 2 × 2 appeared less blurry and preserved most of the small anatomical structures.

### SRR—head-to-head performance of various CNN

Previously published state-of-the-art networks, including 3D SRCNN^1^, 3D FSRCNN^2^, DCSRN^6^, mDCSRN^7^, ReCNN^36^ and MINet^43^ were implemented for performance comparison with TS-RCAN. For the MINet, the official implementation provided by the original authors was adopted. For the other 3D SRR baselines, in the absence of publicly available implementations, the networks were reimplemented according to the architectures and parameter settings described in the original publications. All networks were trained with the identical settings (i.e., epochs, learning rate decay and optimizer).

**Fig. 4:**
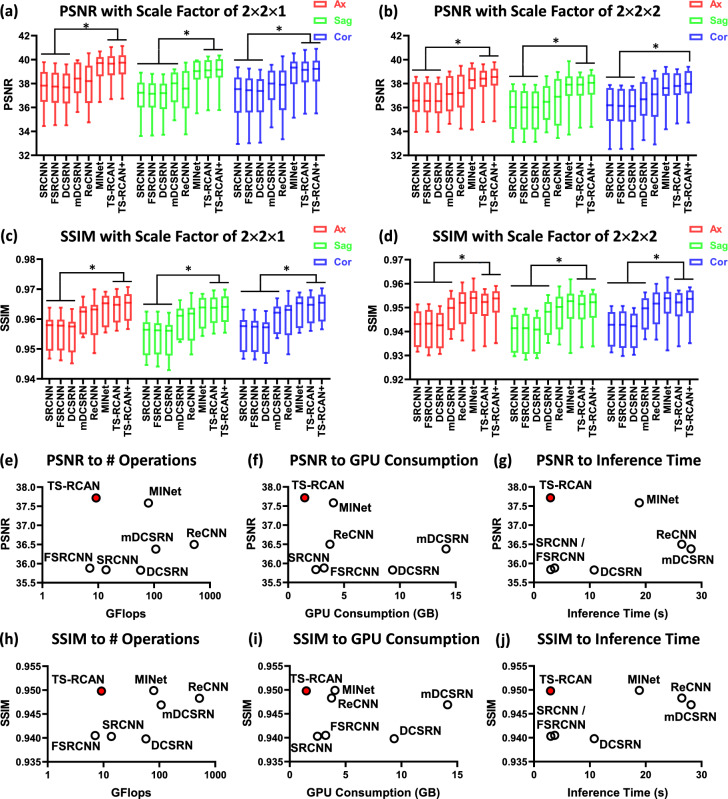
Comparison of TS-RCAN with other state-of-the-art 3D networks in terms of super resolution reconstruction. (**a**)–(**d**) comparison of metrics with two scale factors ($2\times 2\times 1$ and $2\times 2\times 2$), ‘ + ’ represents the SRR with self-ensemble. TS-RCAN achieved comparable performance with MINet and outperformed the other networks. (**e**)-(**j**) Comparison of number of operations, GPU consumption and inference time to PSNR and SSIM with scale factor of $2\times 2\times 2$. TS-RCAN achieved top performance by consuming the minimal computation resources and inference time. * p < *0.05* and ** p < *0.0005*.

For SRR with scale factors of $2\times 2\times 1$ and $2\times 2\times 2$, TS-RCAN outperformed SRCNN, FSRCNN and DCSRN in SSIM and PSNR with significant differences (*p* value range: 0.004 to 0.045; Fig. 4a–d). Although a significant difference was not observed, the mean SSIM/PSNR of TS-RCAN were higher than mDCSRN and ReCNN, and was comparable to MINet which used the similar backbone like TS-RCAN.

Comparing computation resources for $2\times 2\times 2$ SRR shown in Fig. 4e–j, TS-RCAN network demonstrated a similar number of operations to 3D SRCNN and 3D FSRCNN, while being significantly more efficient than other networks. TS-RCAN also consumed less GPU memory and achieved faster inference times than all other networks (*p* value < 0.0001). Comparing to ReCNN / MINet, which had SRR performances closest to TS-RCAN, TS-RCAN consumed 60.4% / 63.5% less VRAM, respectively. The mean inference time of TS-RCAN for processing a full image volume was only 11.2% of ReCNN and 15.7% of MINet. Detailed quantitative results are available in Supplementary Table S1.

**Fig. 5:**
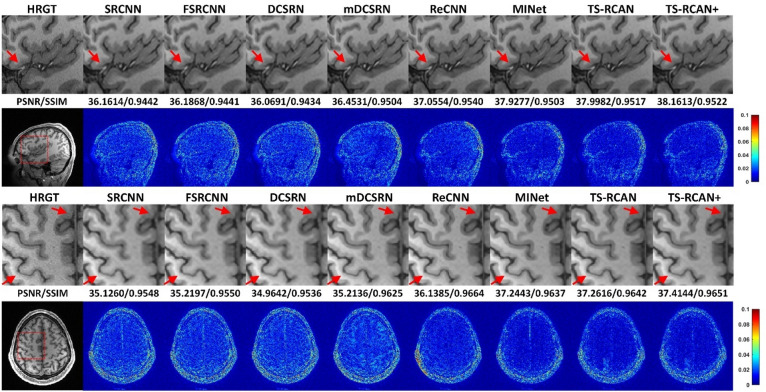
Qualitative comparison of 3D MRI SRR to the state-of-the methods with the scale factor of $2\times 2\times 2$. The SR images of SRCNN, FSRCNN and DCSRN exhibited noticeable blurriness in both the sagittal and axial views, leading to an almost complete loss of gray matter visibility in the hand knob region (indicated by the arrow). In contrast, ReCNN, MINet, and TS-RCAN demonstrated comparable performance with reduced errors and improved differentiation of small anatomical structures (arrow).

In the qualitative comparison of 3D MRI SRR methods with a scale factor of 2 × 2 × 2 shown in Fig. 5 and Supplementary Fig. S3, the SR images reconstructed by SRCNN, FSRCNN, and DCSRN appeared blurry in both sagittal and axial views. This blurriness led to an almost complete loss of gray matter delineation in the hand knob region (indicated by the arrow in the lower row). In contrast, ReCNN, MINet, and TS-RCAN demonstrated significantly improved performance, with reduced errors and better distinguishability of small anatomical structures (arrows in both the upper and lower rows), providing enhanced clarity and anatomical detail.

### Motion artifact reduction – performance of TS-RCAN versus UNet

The MA-corrupted images generated by the proposed periodic MA generation algorithm revealed a mean drop of 0.049 to 0.074 in SSIM and 2.5 to 3.3 dB in PSNR when motion severity increased (by reducing ${T}_{s}$ by 50% each time). The through-plane rotation also resulted in additional decrements of 0.003 to 0.012 in SSIM and 0.4 to 0.5 dB in PSNR compared to only in-plane rotation.

**Fig. 6:**
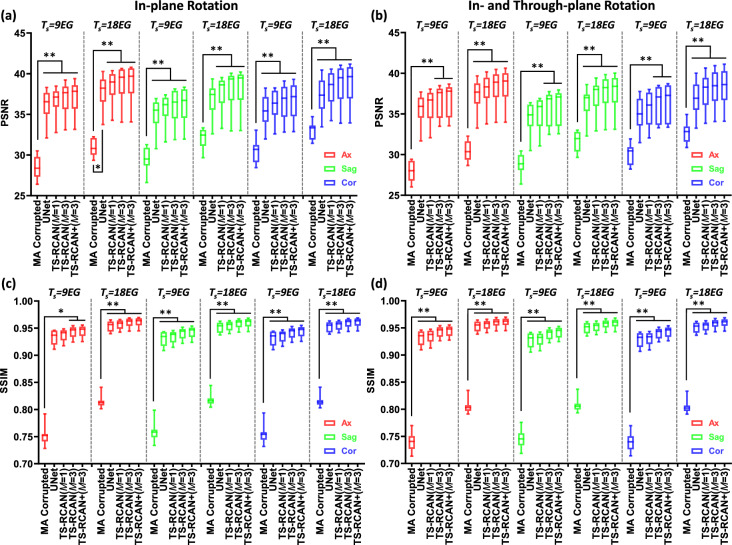
Performance comparison of UNet and TS-RCAN for restoration of image quality under the most severe simulated motion artifact conditions (${T}_{s}=9EG/18EG$). Here, $EG$ (echo group) denotes a fixed number of k-space lines, and ${T}_{s}$ represents the inter-motion interval during which the object remains stationary. Smaller ${T}_{s}$ values (fewer $EGs$) indicate shorter stationary periods, longer relative motion duration, and a larger proportion of k-space lines affected by motion, resulting in more severe artifacts. TS-RCAN with $M=3$ significantly improved image quality in all cases, which was not the case for UNet and TS-RCAN with $M=1$. Moreover, TS-RCAN outperformed UNet in SSIM/PSNR with improvement in all directions and severities of motion artifacts. (**a**)/(**c**) PSNR and SSIM of MA-corrupted and reduced images with 5 degrees in-plane rotation; (**b**)/(**d**) PSNR and SSIM of MA-corrupted and reduced images with 5 degrees in-plane rotation and 5 degrees through-plane rotation. ‘ + ’ represents the MAR with self-ensemble. * p < *0.05* and ** p < *0.0005*.

For all evaluated motion artifact severities, TS-RCAN ($M=3$ , both with and without self-ensemble) significantly improved the mean SSIM / PSNR of MA reduced images compared to the noncorrected data sets (increment of mean SSIM / PSNR range: 0.18 to 0.20 / 6.33 to 8.77 dB; *p* value range: 0.005 to 0.013 for axial direction of ${T}_{s}=9EG$ in Fig. 6c, and ≤ 0.001 for the other cases). This improvement was not observed in the UNet results.

The 2D U-Net was implemented following Wang et al.^24^. The transposed convolution layers used for upsampling were replaced with pixel-shuffle upsampling to avoid potential checkerboard artifacts without altering the overall network design. In direct comparison to UNet, TS-RCAN showed improved performance: the mean SSIM / PSNR increased by over 0.004 / 0.5 dB with $M=1$, and up to 0.014 / 1.48 dB with $M=3$ for ${T}_{s}=9EG$, although no significant difference was detected (*p* value range: 0.22 to > 0.99; Fig. 6). The difference between UNet and TS-RCAN was most pronounced in datasets with the most severe MA. Detailed quantitative results are available in Supplementary Table S2.

**Fig. 7:**
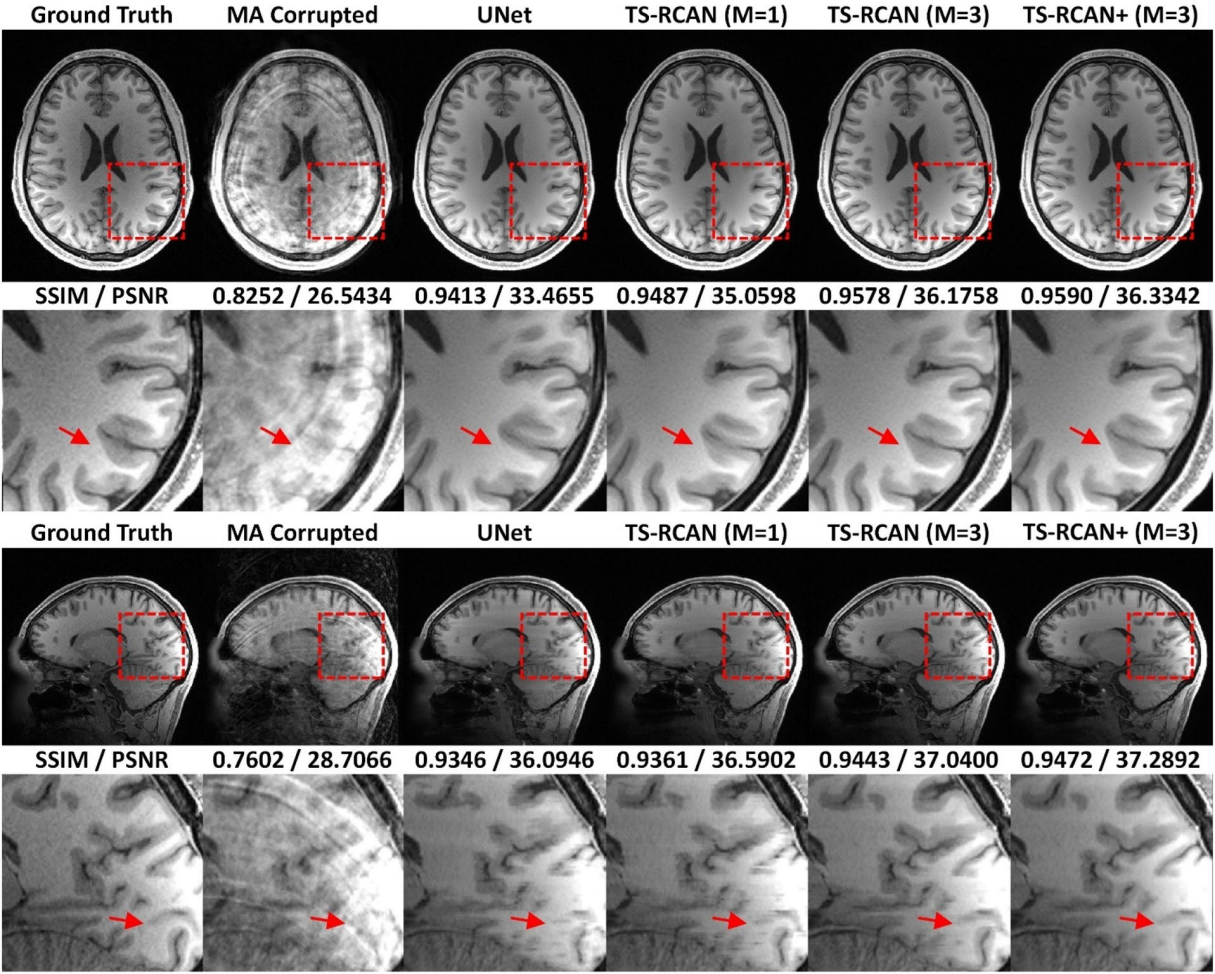
Qualitative comparison on axial and sagittal planes for motion artifact reduction in group with most severe motion artifacts (${T}_{S}=9EG$, 5 degrees in-plane and through-plane rotation).

In the qualitative comparison, the results of the quantitative analysis can be observed, with improved image quality of all networks compared to motion-corrupted source data, and the highest image quality achieved by TS-RCAN + with $M=3$ network (Fig. 7). In the axial plane, the UNet-corrected image contained several incorrectly restored anatomical structures, while the quality of the restored image using TS-RCAN with $M=1$ was improved with reduced errors (red arrow). With $M>1$, TS-RCAN provided significantly improved image quality, with most anatomical structures well represented. The difference between the networks is even greater in the sagittal plane. Due to the lack of through-slice information, the image corrected by UNet contained a severe through-slice mismatch, which was slightly reduced by TS-RCAN with $M=1$ and significantly reduced with $M=3$ (red arrow).

### Uncertainty evaluation

**Fig. 8:**
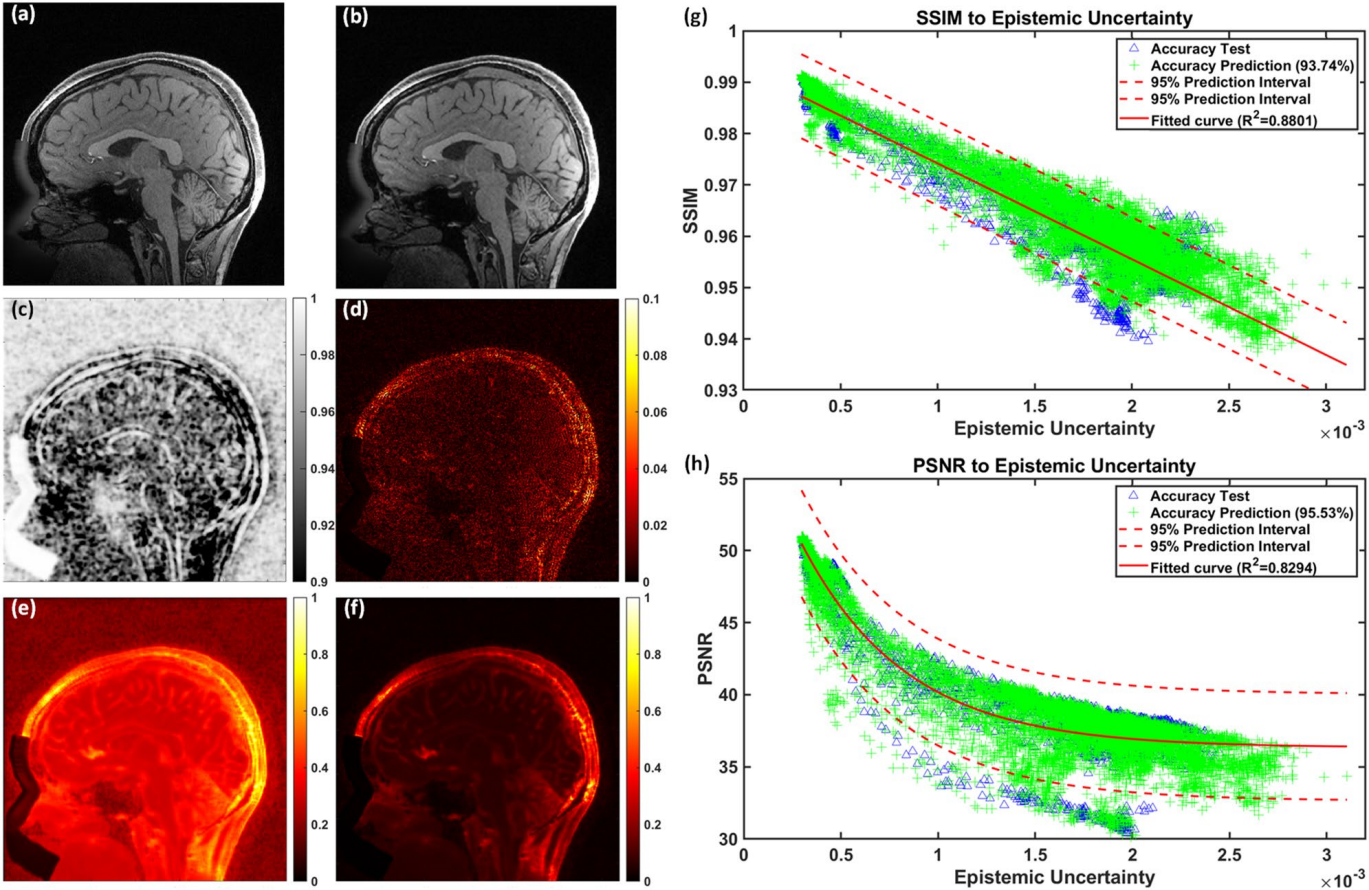
Quantitative and qualitative evaluation of uncertainty. (**a**) GT image; (**b**) SR image; (**c**) SSIM map; (**d**) absolute error map between GT and SR images; (**e**) aleatoric uncertainty map rescaled to the range of 0 to 1 , illustrating its widespread presence across the image volume, particularly in the background where random noise dominates; (**f**) epistemic uncertainty map also rescaled to the range of 0 to 1, emphasizing anatomical structures: regions with high epistemic uncertainty correspond to lower SSIM values and higher absolute error values; (**g**) Linear regression for SSIM and (**h**) exponential regression for PSNR against epistemic uncertainty, using the accuracy test data (blue triangles) achieved ${R}^{2}$ values above 0.8, indicating a strong fit. Additionally, 93.7% and 95.5% of accuracy prediction data (green crosses) fell within the 95% predict intervals of SSIM and PSNR, respectively.

An example of a GT image along with its corresponding SR image, SSIM map, absolute error map, and uncertainty maps is presented in Fig. 8. The aleatoric uncertainty map (Fig. 8e) was distributed throughout the entire image volume, particularly in the background regions where random noise was predominant. In contrast, the epistemic uncertainty map (Fig. 8f) emphasized the anatomical structures, with regions showing high epistemic uncertainty aligning with areas of lower SSIM values (Fig. 8c) and higher errors in the absolute error map (Fig. 8d), and vice versa.

By analyzing the mean epistemic uncertainty of each image slice alongside the corresponding SSIM and PSNR values, strong correlations were identified. As illustrated in Fig. 8g,h, a linear regression analysis was conducted between the mean epistemic uncertainty and SSIM values using data from 10 test patients (represented by blue triangles), resulting in a regression equation (solid red line) with a 95% prediction interval (region between dashed red lines). For PSNR, due to its logarithmic nature, an exponential regression was applied, also yielding a regression equation with a 95% prediction interval. Both regression models demonstrated good fit with ${R}^{2}$ values exceeding 0.8. To validate the predictive accuracy of these regression models, an additional 40 datasets independent from the training, validation, and test groups were used. These datasets, shown as green crosses in Fig. 8g,h, exhibited strong alignment with the prediction intervals, with 93.7% and 95.5% of the data points falling within the respective intervals for SSIM and PSNR, confirming that the correlations between mean epistemic uncertainty and SSIM/PSNR align closely with the predicted distributions.

### Generalization to an independent dataset (MR-ART)

To further evaluate the generalization capability of the proposed method beyond the HCP dataset, we conducted additional experiments on the publicly available MR-ART dataset. In this evaluation, motion-free (“Standard”) MR-ART images were used for super-resolution testing after down-sampling. Although both MR-ART and HCP provide T1-weighted images acquired at 3 T using MPRAGE-based protocols, they differ substantially in several key aspects. MR-ART data were acquired on a Siemens Prisma scanner with a 20-channel head–neck coil, whereas HCP uses a dedicated Connectome Skyra system with a 32-channel head coil, resulting in distinct coil sensitivity profiles and SNR characteristics. In addition, differences in scanner hardware and acquisition chains, including gradient performance, B₀/B₁ field properties, as well as the spatial resolution (1.0 mm isotropic for MR-ART versus 0.7 mm isotropic for HCP), lead to systematic shifts in image appearance and spatial frequency content. Taken together, these factors make MR-ART a meaningful out-of-distribution (OOD) dataset relative to the models trained on HCP data.

**Fig. 9:**
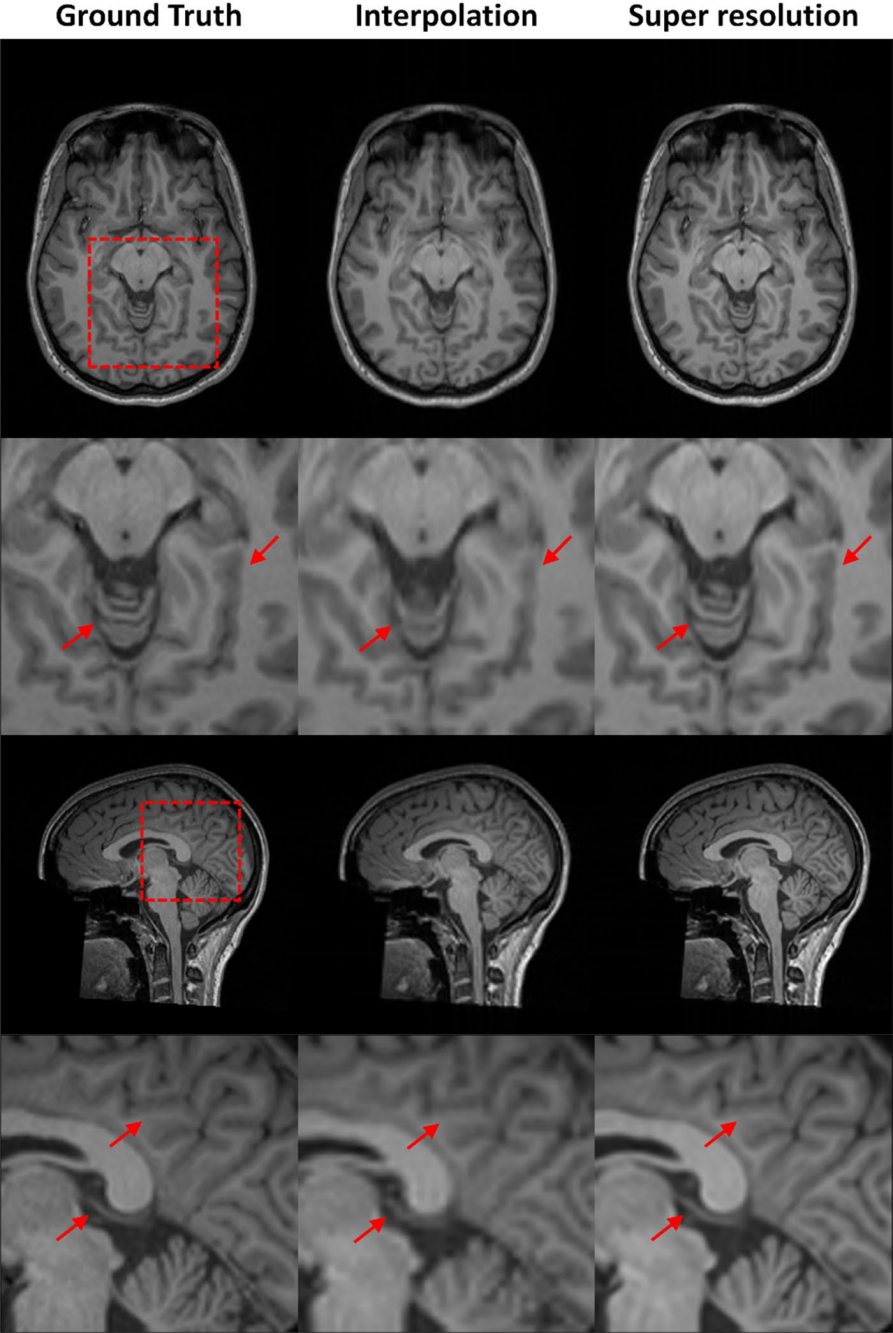
Super resolution reconstruction on MR-ART dataset with qualitative comparison to tricubic interpolation. Tricubic interpolation results exhibit pronounced blurring and loss of fine anatomical details, with white–gray matter boundaries appearing poorly defined. In contrast, the SRR images produced by the proposed method show a substantial improvement in image sharpness, recovering fine structural details and yielding markedly sharper and more distinct tissue boundaries. These results highlight the advantage of learning-based super-resolution over conventional interpolation, even under out-of-distribution conditions.

We first evaluated the super-resolution performance of the proposed method on the MR-ART dataset as an OOD test. For the $2\times 2\times 1$ and $2\times 2\times 2$ down-sampling configurations, the proposed method achieved SSIM values of 0.9633 and 0.9207, and PSNR values of 39.4979 dB and 34.3336 dB, respectively. As expected, these values are lower than those obtained on the HCP dataset, reflecting the substantial domain shift between the two datasets. However, these results were obtained without any retraining or fine-tuning on MR-ART, and still demonstrate strong reconstruction quality under cross-dataset generalization. Compared with tricubic interpolation, the SRR results are visibly sharper and exhibit markedly improved recovery of anatomical details (Fig. 9). In particular, tissue boundaries and fine structures that appear blurred or partially merged in the interpolated images are better preserved in the SR reconstructions, highlighting the benefit of learning-based super-resolution even under OOD conditions.

**Fig. 10:**
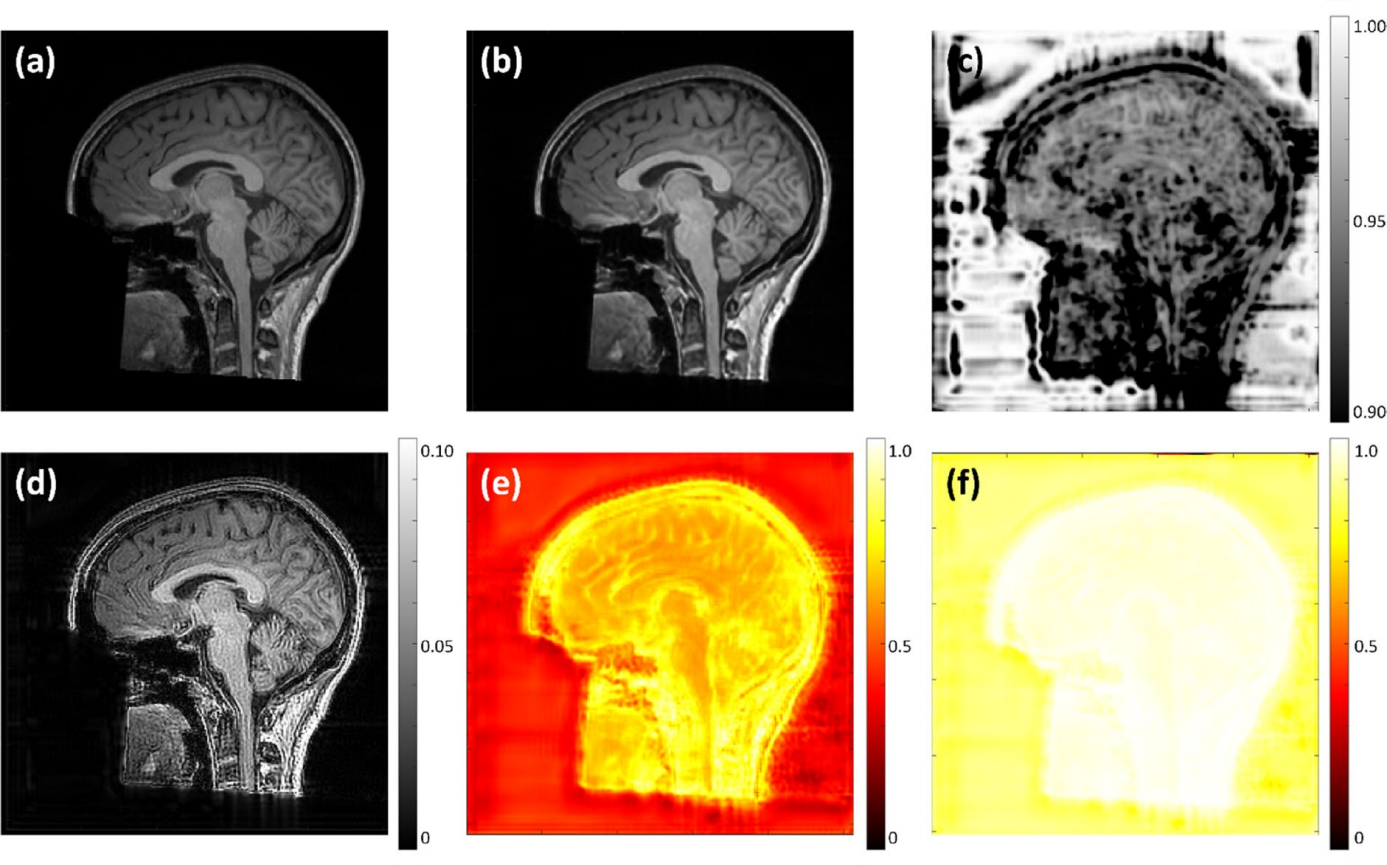
Uncertainty estimation of MR-ART. (**a**) GT image; (**b**) SR image; (**c**) SSIM map; (**d**) absolute error map between GT and SR images; (**e**) aleatoric uncertainty map rescaled to the range of 0 to 1, the globally elevated values across the image volume illustrate the distribution difference between HCP and MR-ART datasets; (**f**) epistemic uncertainty map rescaled to the range of 0 to 1 still corresponds to SSIM and absolute error maps.

The corresponding uncertainty maps in Fig. 10 exhibit elevated values across large portions of the image volume, in contrast to the lower and more spatially localized uncertainty observed on HCP data. This behavior is consistent with the pronounced distribution shift between two datasets: the model was only trained on HCP data and is therefore exposed to feature distributions that differs substantially from those of MR-ART. As a result, the increased uncertainty reflects reduced model confidence when operating with data outside the training distribution. Importantly, the spatial patterns of the error maps, SSIM maps, and uncertainty maps remain consistent with those observed on HCP data. Owing to the complete absence of MR-ART samples during training, the aleatoric uncertainty maps exhibit globally elevated values, indicating a substantial mismatch between the data distributions of MR-ART and HCP. In contrast, epistemic uncertainty remains spatially structured, with higher values concentrated in regions containing anatomical structures, where it continues to correspond to areas of increased reconstruction error and reduced SSIM. Although epistemic uncertainty values in background regions are also elevated relative to HCP, they remain clearly lower than those in structurally complex regions, preserving the distinction between anatomically informative and homogeneous areas.

For MAR, the motion-corrupted images (Motion 1 and Motion 2) of MR-ART dataset were used to assess the performance through qualitative comparison with the corresponding standard images. In addition to domain differences, MR-ART provides images corrupted by real subject motion acquired during scanning. Participants were instructed to move during predefined motion windows; however, the exact timing, velocity, duration, and rotation angle of the motion within each window were neither constrained nor measured. As a result, motion realizations vary substantially across repetitions and participants and are inherently non-reproducible, making the motion patterns closely resemble natural head motion encountered in routine clinical MRI examinations.

**Fig. 11:**
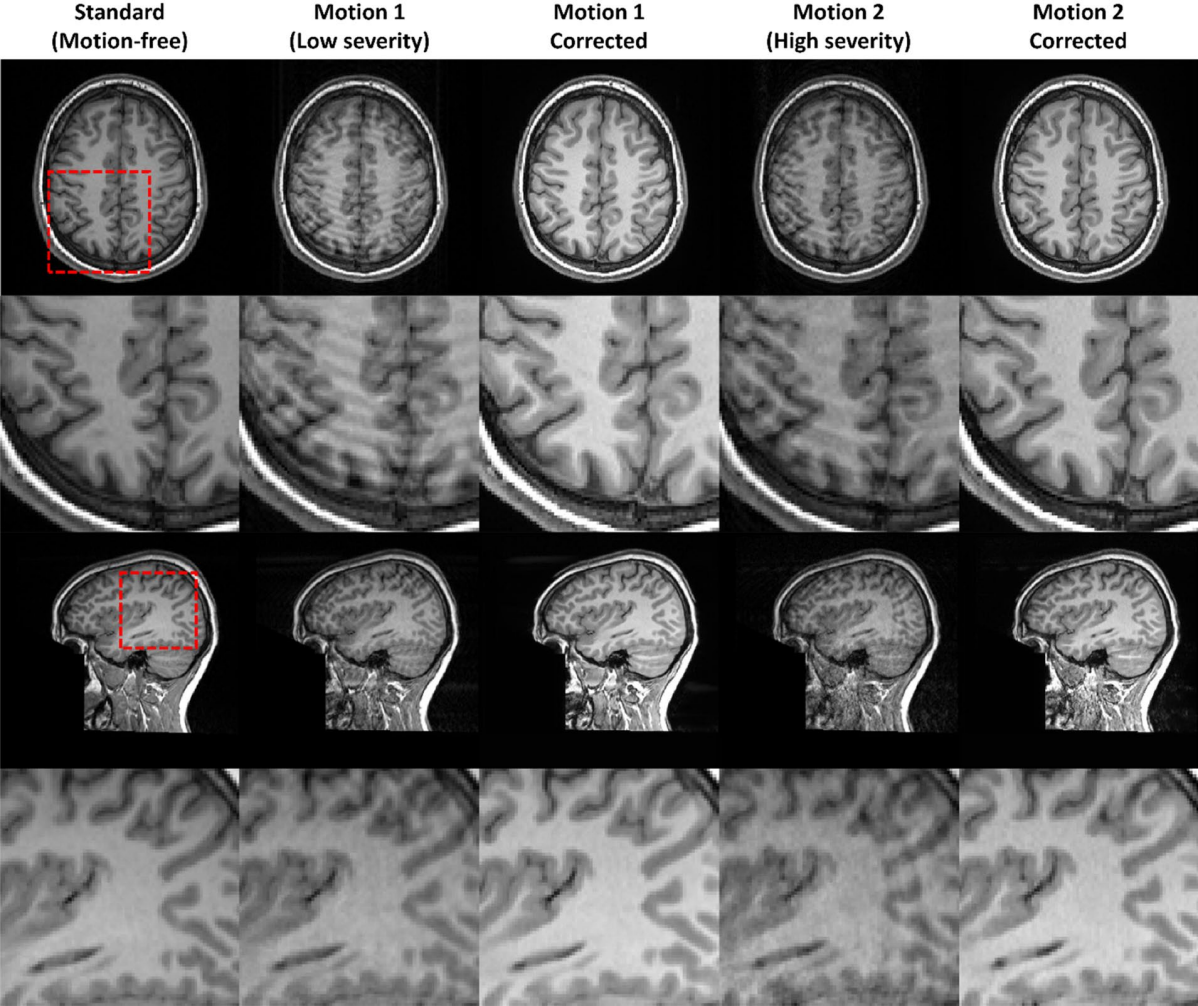
Performance motion artifact reduction to real subject motion on the MR-ART dataset. Five image types are shown: a standard (motion-free) image, Motion 1 with low artifact severity, Motion 1 after correction, Motion 2 with high severity, and Motion 2 after correction. For each type, axial (in-plane) views and sagittal (through-plane) views with zoomed-in regions are displayed. Because no image registration was performed between the standard and motion-corrupted images, the shown slices represent closely matched but not identical anatomical locations.

Since the motion-corrupted and reference (“Standard”) images cannot be reliably aligned in high-precision with registration, which precludes meaningful voxel-wise quantitative metrics. We therefore focus on visual comparison to assess artifact suppression and structural fidelity. Qualitative results in Fig. 11 show that the model trained on HCP data with simulated motion artifacts is able to effectively reduce real motion-induced artifacts in MR-ART, despite the substantial domain shift and the absence of retraining. In the majority of examples, prominent motion-induced artifacts are substantially suppressed, while tissue contrast and anatomical structures are well preserved. Minor residual artifacts and only mild over-smoothing effects are observed in most cases, and blurriness only in extreme cases (Supplementary Fig. S4). The overall signal intensity increase observed in the corrected images is attributable to the domain shift between the HCP and MR-ART datasets, which differ substantially in their global signal intensity distributions and contrast characteristics.

## Discussion

A GPU-efficient, unified 2D deep neural network (TS-RCAN) was adapted for pseudo-3D MRI super-resolution reconstruction and motion artifact reduction, resulting in superior image quality. The performance of TS-RCAN was equal to or better compared to previous SRR or MA reduction CNNs. In contrast to previously published 3D convolutional neural networks, the advanced performance of TS-RCAN for SRR was not dependent on high GPU workload and long inference time. Furthermore, the evaluation of the pixel-wise uncertainty of the TS-RCAN network yielded robust accuracy values. These results are of clinical importance as acquisition time and impact of motion are critical factors in clinical MRI protocols, especially in high-resolution 3D sequences. Consequently, both features of this network combined with its high accuracy may improve image quality and interpretability, and potentially support improved diagnostic accuracy in clinics across various MRI applications.

This study has several strengths. First, this study comprehensively analysed the impact of various down-sampling factors and identified when $M>1$ with $1\times 1\times 2$ for $\times 2$ acceleration and $2\times 2\times 2$ for $\times 4$ acceleration results in the best SRR performance. Self-ensemble further improved the performance of the network without any additional operations. Therefore, these down-sampling factors are recommended in deep learning-based SRR to fit into existing clinical workflows. In particular, the down-sampling in slice direction (e.g., $1\times 1\times 2$) is highly relevant to clinical practice, as many MRI protocols employ anisotropic resolution with slice thickness larger than in-plane resolution. Since isotropic resolution is generally preferred for diagnostic evaluation and multiplanar reconstruction, large slice thickness followed by super-resolution reconstruction provides a practical pathway toward achieving isotropic high-resolution images while reducing acquisition time. This strategy therefore aligns well with common clinical acquisition patterns and diagnostic preferences.

Second, a combined CNN for SRR and MA reduction was developed. Third, the performance of the novel CNN was directly compared with existing CNNs in a head-to-head comparison. Regarding SRR, we confirmed the results of Chen et al.^6,7^, Pham et al.^8^, and Koktzoglou et al.^9^, who reported that 3D SRR outperformed 2D SRR. TS-RCAN significantly outperformed 3D SRCNN^1^, 3D FSCNN^2^ and DCSRN^6^ in 3D SRR by over 0.01 in SSIM and 1.9 dB in PSNR with scale factor of $1\times 1\times 2$, and over 0.01 in SSIM and 1.5 dB with scale factor of $2\times 2\times 2$. TS-RCAN also outperformed mDCSRN^7^ and ReCNN^36^, although significant difference was not detected. TS-RCAN showed comparable performance with MINet^43^, which had the same backbone network.

Regarding the MAR, TS-RCAN significantly outperformed 2D UNet^23,24^, particularly with the improved through-slice agreement. Unlike previous studies that employed random motion patterns for retrospective MA generation^25–27^, our approach utilized a predefined movement pattern with adjustable movement duration and frequency, allowing for controlled and quantifiable severity. By modifying the motion frequency, our experiments produced MA-corrupted images with SSIM and PSNR values that increased linearly, ensuring a systematic assessment of artifact severity. The motion pattern can be modified based on any specific scenario.

Several methods using 2D neural networks to reconstruct 3D MR images were proposed in previous studies, but each had drawbacks. Du et al. proposed to interpolate the LR image to the expected size before feeding the 2D network slice by slice^44^. However, this method still reconstructed 2D images without fusing the features from multiple slices in an image volume. Zhang et al. proposed to use 2D network to reconstruct the in-plane SR images in three orthogonal planes, and use another 2D network to combine the three groups of reconstructed SR images^45,46^. Sood and Rusu et al. proposed a similar method reconstructing 2D slices in two orthogonal planes and then rebuilding the 3D image volumes^10^. These methods highly increased the complexity and demands several times of computation resources of single 2D networks. Georgescu et al. proposed to process the 3D image volume with two networks progressively^12^. A 2D network was used to process the images slice by slice for in-plane SRR, and a 3D network for through-plane SRR.

In contrast, we propose to use a single 2D network with single step of processing to reconstruct 3D image slabs. Regarding the consumption of GPU resource and inference time, TS-RCAN consumed comparable GPU resource and inference time with 3D SRCNN and 3D FSRCNN, down to 15.8% / 10.5% / 39.6% / 36.5% of GPU resources and less than 27.4% / 10.2% / 11.2% / 15.7% of inference time compared to DCSRN^6^ / mDCSRN^7^ / ReCNN^36^ / MINet^43^. Thus, it can be easily deployed on any consumer GPU and reconstruct the image very timely.

In addition, we adopted evidential regression learning to estimate the uncertainty maps simultaneously with restoring images. Qin et al. has demonstrated the uncertainty map to predict the accuracy of reconstructed super-resolution images^29^, but their method was not able to distinguish different sources of uncertainty. In contrast, the method adopted in this manuscript separated the aleatoric and epistemic uncertainties. Experimental results revealed that the aleatoric uncertainty highly depended on the noise from the training data. Besides, the epistemic uncertainty map corresponded to the absolute error map and the SSIM map between the ground truth and the restored image. In addition, we investigated the correlation of the epistemic uncertainty to the SSIM/PSNR values. Our experiments revealed that the mean epistemic uncertainty of each image slice appeared linearly and exponentially related to the SSIM and PSNR, respectively. Thus, even when ground truth is unavailable in clinical settings, the SSIM and PSNR values can be predicted using the regression equations. Besides, the epistemic uncertainty map could also help doctors to identify which regions of the reconstructed SR images are more reliable, and avoid mis-guided diagnosis or treatments caused by incorrectly generated contents.

Finally, the additional evaluation on the MR-ART dataset highlights both the generalization capability and the limitations of the proposed method under substantial domain shift. Despite being trained exclusively on HCP data, the model generalizes well to MR-ART for both super-resolution and motion artifact reduction without retraining, including realistic, non-deterministic subject motion. This suggests that the proposed framework is not tied to a specific scanner, coil configuration, or simulated degradation model. At the same time, uncertainty maps on MR-ART exhibit globally elevated values compared with in-distribution data, which is consistent with the uncertainty formulation used in this work. Because MR-ART lies largely outside the feature distribution observed during training, both aleatoric and epistemic uncertainty increase, reflecting reduced model confidence rather than reconstruction failure. These results emphasize that uncertainty-based quality assessment is most informative within, or close to, the training distribution, while under strong domain shift it primarily serves to flag out-of-distribution inputs.

We acknowledge several limitations. First, although we included additional experiments on an independent dataset, the current study remains focused on T1-weighted images of the brain, and the proposed framework has not yet been evaluated on other clinically important contrasts (e.g., T2, FLAIR), pathological cases, or additional anatomical regions. Extending the evaluation to such scenarios is necessary to fully assess robustness across the broader spectrum of clinical MRI applications. Second, although MR-ART provides real subject motion with a degree of randomness, the motion still follows a controlled protocol. Motion encountered in routine clinical practice may be more heterogeneous and unpredictable, motivating further validation on more representative clinical datasets. Furthermore, the network was trained on a limited set of simulated motion artifact patterns, which cannot fully represent the diversity of artifact characteristics encountered in real-world MRI. In particular, motion types involving translational movement (drift movements) were not included in the simulated training model. When motion severity or artifact structure falls outside the feature distribution observed during training, the model may produce local structural inaccuracies or hallucinated details during motion artifact reduction. In particular, the domain gap between simulated motion artifacts and complex real motion patterns may lead to more pronounced failure modes in extreme cases. Although promising performance and accuracy were observed, further studies in patient cohorts are required for a comprehensive assessment of clinical utility.

Another limitation of this study is that the employed TS-RCAN did not incorporate some of the most recent modules, such as Mamba or transformer-based components. These modules are known to improve image reconstruction, but their direct extension to 3D networks remains highly challenging due to the excessive computational cost. To ensure computational feasibility, most existing 3D networks also adopt relatively simplified architectures. In our case, we intentionally chose a comparatively basic CNN backbone to more clearly highlight the intrinsic advantage of the proposed pseudo-3D framework. Importantly, the pseudo-3D design is fully compatible with modern modules including Mamba and transformer, which could be integrated in future work to further improve reconstruction quality while still maintaining a low computational burden.

## Conclusion

In conclusion, a time and GPU-efficient unified deep neural network framework based on 2D CNN for 3D SRR and MAR is proposed. The $1\times 1\times 2$ down-sampling factors for $\times 2$ acceleration and $2\times 2\times 2$ for $\times 4$ acceleration were identified as optimal. TS-RCAN outperformed the 3D networks of DCSRN, mDCSRN, and ReCNN in SRR, and outperforms UNet in MAR, in SSIM/PSNR performance, GPU load and interference time. Additionally, TS-RCAN provided the uncertainty information, which can be used to estimate the quality of the reconstructed images, enhancing safety under clinical settings.

## Supplementary Information

Supplementary Information.

## Data Availability

The MRI data analyzed in this study are publicly available from the Human Connectome Project (HCP-YA) at https://www.humanconnectome.org/. Access requires free registration and agreement to the HCP Data Use Terms. The MR-ART dataset is publicly available under a CC0 license at https://openneuro.org/datasets/ds004173/versions/1.0.2. The source code for this study is available at: https://github.com/HaoLiMRI/TS-RCAN. All derived data generated in this study through the analysis and processing of these MRI datasets are available from the corresponding author upon reasonable request.
