# Supplementary material for: Performance of a GPU- and time-efficient pseudo-3D network for magnetic resonance image super-resolution and motion artifact reduction
Source: Sci Rep. 2026 Mar 21;16:9654. doi: 10.1038/s41598-026-43804-1 (PMC13009481; doi:10.1038/s41598-026-43804-1)
Supplement: Supplementary file 1 — Supplementary Information. [file 41598_2026_43804_MOESM1_ESM.pdf]

Performance of a GPU- and Time-Efficient Pseudo-3D Network for Magnetic Resonance Image Super-Resolution and Motion Artifact Reduction

Authors:

Hao Li[1], Jianan Liu[2], Marianne Schell[1], Tao Huang[3], Arne Lauer[1], Katharina Schregel[1,4], Jessica Jesser[1], Dominik F Vollherbst[1], Martin Bendszus[1], Sabine Heiland[1], Tim Hilgenfeld[1,*]

Author Institutions:

[1] Department of Neuroradiology, University Hospital Heidelberg, Heidelberg, Germany

[2] Momoni AI, Gothenburg, Sweden

[3] College of Science and Engineering, James Cook University, Smithfield, Australia

[4] Department of Radiology, Section Neuroradiology, Jena University Hospital, Jena, Germany

*** Corresponding Author:**

Tim Hilgenfeld, MD
Heidelberg University Hospital
Department of Neuroradiology
Im Neuenheimer Feld 400
69120 Heidelberg
Germany

Email: tim.hilgenfeld@med.uni-heidelberg.de | Phone: +49 6221 56 7566 | Fax: +49 6221 56 4673

Conflict of interest:

There is no relevant conflict of interest or financial industry support of the project.

Data Availability Statement:

Research data is available upon reasonable request.

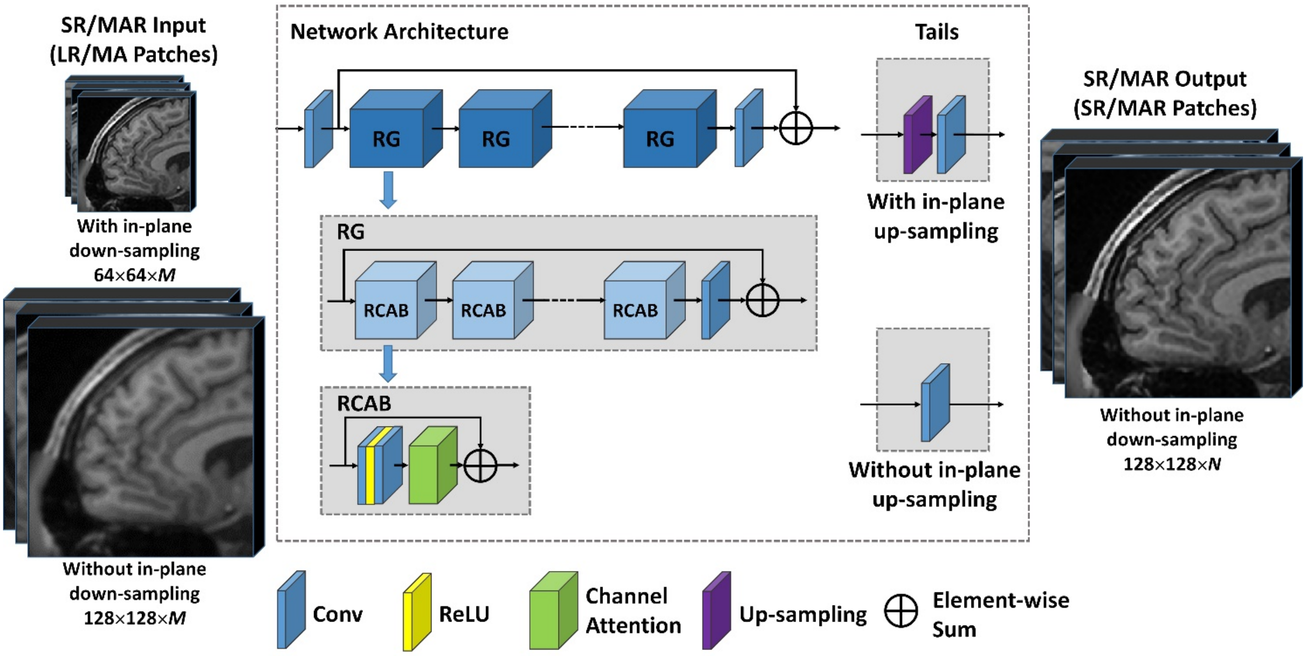

Supplementary Figure S1 Pipeline of thin-slab RCAN (TS-RCAN) used for MRI super resolution reconstruction and motion artifact reduction.

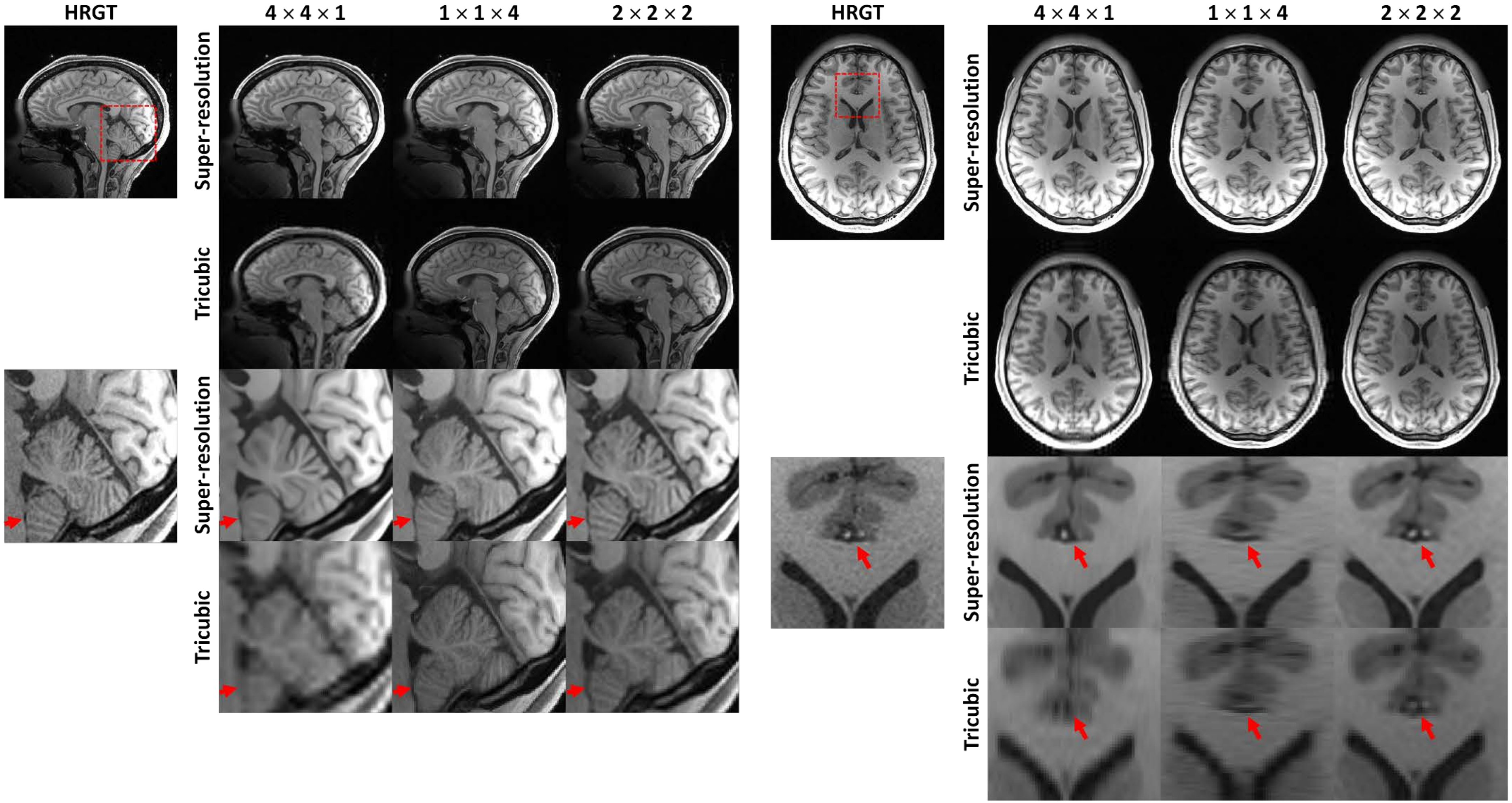

Supplementary Figure S2 Comparison between super-resolution reconstruction and tricubic interpolation. Qualitative comparisons in both sagittal (in-plane) and axial (through-plane) views demonstrate the clear advantage of SRR over tricubic interpolation. Interpolated images are highly blurred, with poorly defined tissue boundaries and substantial loss of fine anatomical details. For the 1×1×4 down-sampling case, the interpolated sagittal view may appear visually sharp because no in-plane down-sampling is applied and only through-plane resolution is reduced. However, from a reconstruction standpoint, this interpolation is inherently inconsistent

with the ground truth, as no information is recovered for the missing through-plane content. This inconsistency becomes evident in the corresponding axial views, which reveal substantial errors along the through-plane direction. In contrast, SRR reconstructs fine anatomical structures and produces sharper images with clearly delineated tissue boundaries in both in-plane and through-plane orientations.

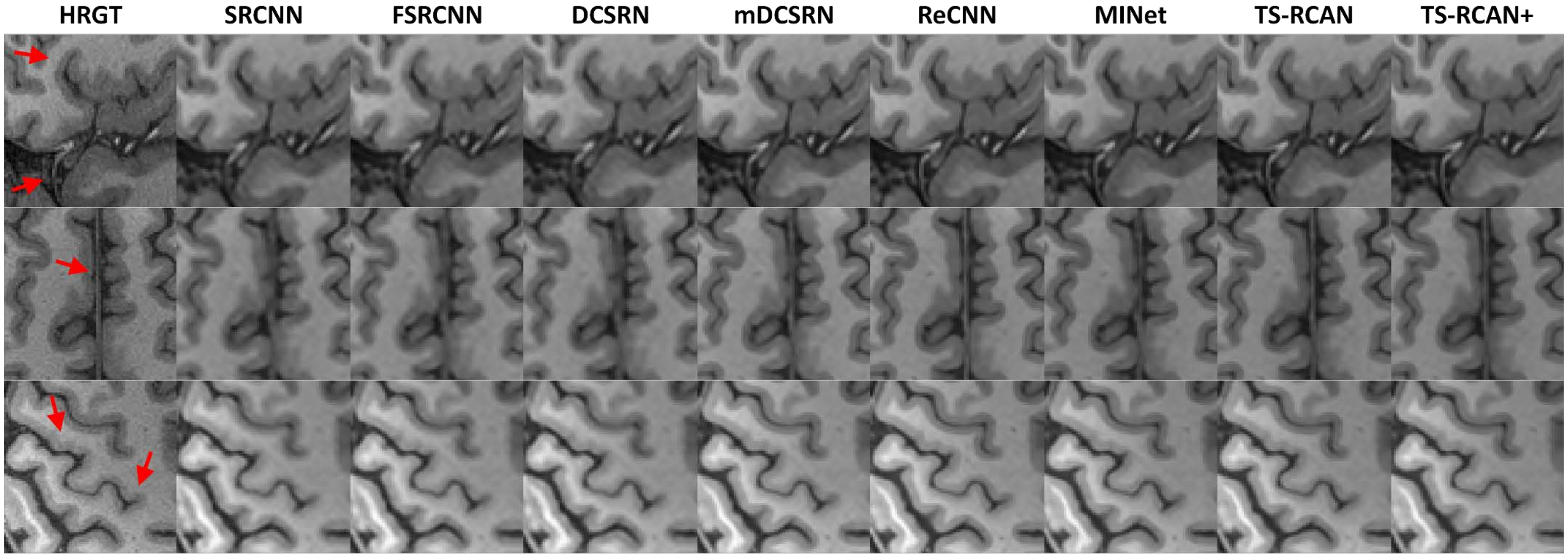

Supplementary Figure S3 Zoomed-in qualitative comparison with state-of-the-art methods. Magnified views are shown to highlight differences in the reconstruction of fine anatomical details. In the first row (sagittal view), only ReCNN, MINet, and TS-RCAN are able to correctly reconstruct small vascular and other fine-scale structures, whereas these details are blurred or missing in other networks. In the second row (axial view), the falx cerebri located at the midline is clearly delineated only by ReCNN and TS-RCAN, indicating that TS-RCAN achieves through-plane information recovery comparable to that of 3D network with substantially larger model capacity. In the first and third rows, gray-white matter differentiation appears markedly blurred or indistinguishable in SRCNN, FSRCNN, and DCSRN, while clear tissue contrast is preserved in ReCNN, MINet, and TS-RCAN.

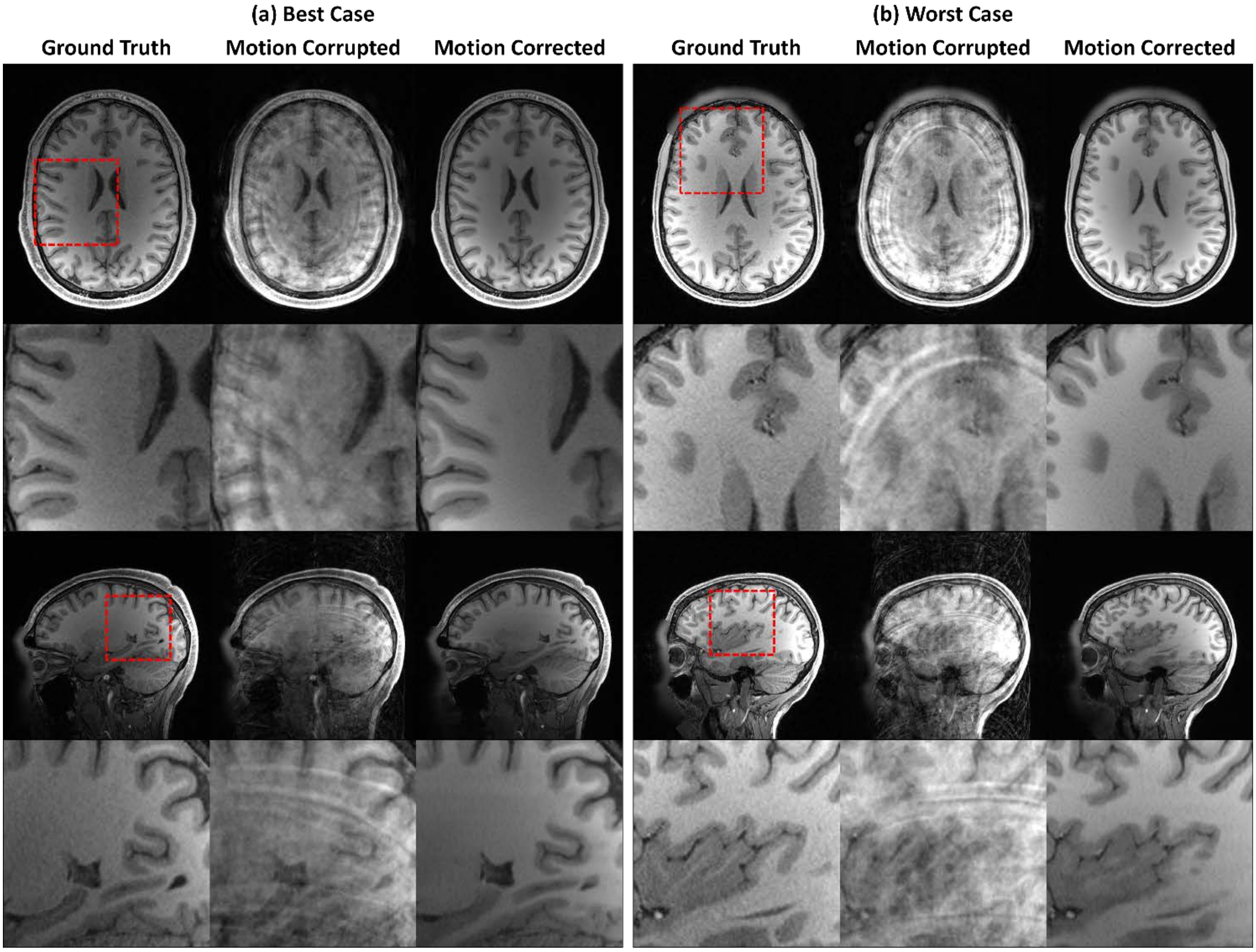

Supplementary Figure S4 Best- and worst-case examples of motion artifact reduction on the HCP dataset. Best- and worst-case examples are selected based on the corresponding SSIM and PSNR values. For comparison, the performance gap between best- and worst-case examples observed on the HCP dataset is relatively small. In both cases, the proposed method effectively suppresses motion artifacts while preserving the majority of anatomical structures, in both in-plane and through-plane views. Minor inaccuracies in localized structures and mild smoothing effects can be observed in some regions during artifact removal, but overall structural fidelity is largely maintained.

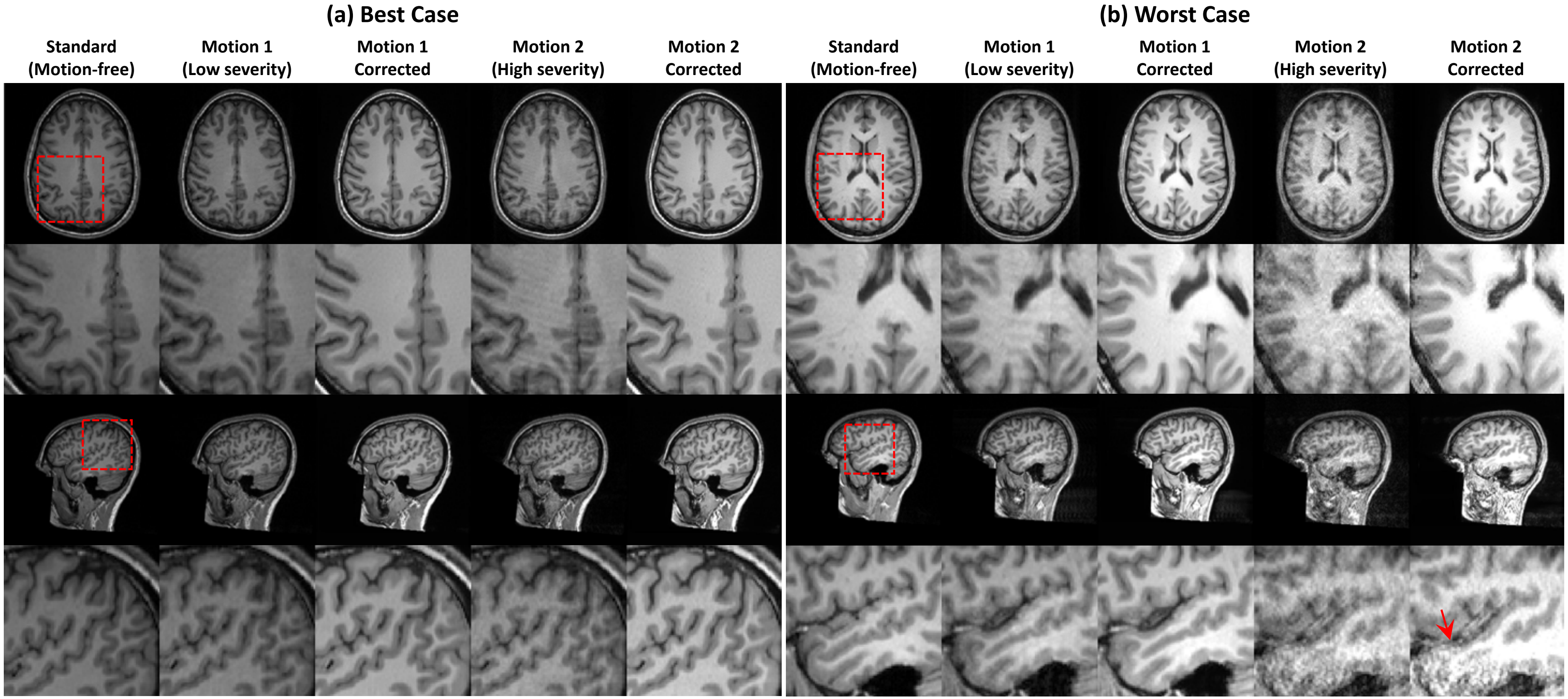

Supplementary Figure S5 Best- and worst-case examples of motion artifact reduction on the MR-ART dataset. The network was trained on the HCP dataset using simulated motion artifacts and applied directly to MR-ART images containing real subject motion without retraining. Five image types are shown: a standard (motion-free) image, Motion 1 (low-severity), Motion 1 after correction, Motion 2 (high-severity), and Motion 2 after correction. For each type, axial (in-plane) and sagittal (through-plane) views with zoomed-in regions are displayed. (a) In the best-case example, motion-induced artifacts are effectively suppressed, resulting in clearer images with improved tissue contrast. (b) In the worst-case example, where motion artifacts are extremely severe and exceed the range encountered during training, the corrected image exhibits noticeable blurring. Such cases represent rare, extreme conditions and highlight the limits of correction under exceptionally strong motion corruption. The arrow highlights a possible hallucination artifact caused by motion patterns not observed during training. Because no image registration was performed between the standard and

motion-corrupted images, the shown slices represent closely matched but not identical anatomical locations.

Supplementary Table S1 Quantitative comparison of TS-RCAN with other state-of-the-art 3D networks in terms of super resolution reconstruction. The best results are highlighted in bold, and the second-best underlined. Inference time corresponds to processing all patches of a 3D image volume, while training time corresponds to 50 training epochs.

Model	Scale Factor	# Ops (GFlops)	GPU Consumption (GB)	Inference Time (s)	Training Time (h)	Axial SSIM / PSNR	Sagittal SSIM / PSNR	Coronal SSIM / PSNR
**BiCubic**		N.A.	N.A.	N.A.	N.A.	0.9218±0.0071 / 34.09±1.23	0.9192±0.0072 / 33.24±1.18	0.9210±0.0071 / 33.18±1.21
**3D SRCNN**		13.841	2.48	3.00	8.7	0.9553±0.0060 / 37.49±1.71	0.9540±0.0062 / 36.83±1.78	0.9550±0.0059 / 36.81±2.07
**3D FSRCNN**		7.079	3.22	3.68	9.5	0.9553±0.0061 / 37.53±1.75	0.9540±0.0062 / 36.87±1.82	0.9550±0.0060 / 36.86±2.11
**DCSRN**		57.50	9.35	10.84	22.8	0.9548±0.0061 / 37.45±1.70	0.9535±0.0063 / 36.80±1.77	0.9545±0.0060 / 36.78±2.04
**mDCSRN**	$2 \times 2 \times 1$	107.54	14.13	28.16	57.6	0.9604±0.0051 / 38.22±1.52	0.9591±0.0053 / 37.59±1.57	0.9602±0.0050 / 37.64±1.81
**ReCNN**		522.74	3.74	26.48	80.5	0.9603±0.0070 / 37.96±1.99	0.9591±0.0070 / 37.27±2.11	0.9601±0.0069 / 37.36±2.36
**MINet**		79.05	3.83	38.74	133.3	0.9635±0.0051 / 39.27±1.41	0.9625±0.0053 / 38.75±1.44	0.9633±0.0051 / 38.78±1.61
**TS-RCAN**		9.12	1.48	5.93	14.9	0.9638±0.0049 / 39.37±1.35	0.9631±0.0051 / 38.87±1.36	0.9637±0.0049 / 38.92±1.53
**TS-RCAN+**		9.12	1.48	5.93	14.9	**0.9644±0.0049** / **39.44±1.37**	**0.9634±0.0051** / **38.94±1.38**	**0.9643±0.0049** / **38.99±1.55**
**Tricubic**		N.A.	N.A.	N.A.	N.A.	0.8977±0.0075 / 32.83±0.92	0.8953±0.0080 / 31.52±1.12	0.8970±0.0076 / 31.81±0.73
**3D SRCNN**		13.841	2.48	3.03	8.7	0.9420±0.0080 / 36.64±1.52	0.9403±0.0081 / 35.84±1.66	0.9418±0.0077 / 35.95±1.81
**3D FSRCNN**		7.079	3.22	3.71	9.5	0.9422±0.0074 / 36.66±1.54	0.9405±0.0075 / 35.88±1.67	0.9420±0.0071 / 35.99±1.83
**DCSRN**		57.50	9.35	10.79	22.8	0.9415±0.0074 / 36.61±1.52	0.9398±0.0075 / 35.83±1.66	0.9413±0.0072 / 35.92±1.81
**mDCSRN**	$2 \times 2 \times 2$	107.54	14.13	28.12	57.6	0.9486±0.0069 / 37.10±1.50	0.9469±0.0071 / 36.38±1.64	0.9484±0.0067 / 36.47±1.77
**ReCNN**		522.74	3.74	26.47	80.5	0.9499±0.0077 / 37.27±1.77	0.9483±0.0077 / 36.50±1.94	0.9498±0.0075 / 36.69±2.12
**MINet**		79.14	4.05	18.86	133.9	0.9512±0.0090 / 38.10±1.61	**0.9499±0.0092** / 37.58±1.70	0.9512±0.0089 / 37.67±1.52
**TS-RCAN**		9.19	1.48	2.97	7.6	0.9501±0.0078 / 38.16±1.39	0.9491±0.0079 / 37.57±1.37	0.9499±0.0078 / 37.64±1.35
**TS-RCAN+**		9.19	1.48	2.97	7.6	**0.9514±0.0078** / **38.30±1.42**	0.9498±0.0080 / **37.72±1.41**	**0.9513±0.0077** / **37.79±1.38**

Supplementary Table S2 Quantitative comparison of TS-RCAN and UNet in terms of motion artifact reduction. The best results are highlighted in bold, and the second-best underlined.

Degrees of Rotation	T_s	Slice Orientation	MA Corrupted SSIM / PSNR	UNet SSIM / PSNR	TS-RCAN (*M*=1) SSIM / PSNR	TS-RCAN (*M*=3) SSIM / PSNR	TS-RCAN+ (*M*=3) SSIM / PSNR
In-plane :5° Through-plane: 0°	$9EG$	Axial	0.7515±0.0169<br>28.42±1.32	0.9334±0.0131<br>36.15±1.94	0.9378±0.0105<br>36.67±1.75	0.9434±0.0100<br>37.07±1.91	**0.9447±0.0100<br>37.19±1.95**
		Sagittal	0.7584±0.0170<br>29.46±1.41	0.9308±0.0130<br>34.93±1.93	0.9346±0.0105<br>35.68±1.83	0.9407±0.0100<br>36.15±2.06	**0.9431±0.0100<br>36.30±2.11**
		Coronal	0.7540±0.0167<br>30.47±1.36	0.9318±0.0128<br>35.49±1.97	0.9359±0.0103<br>36.21±1.85	0.9421±0.0097<br>36.66±2.09	**0.9443±0.0098<br>36.81±2.14**
	$18EG$	Axial	0.8142±0.0107<br>30.95±1.10	0.9547±0.0092<br>37.81±1.93	0.9573±0.0083<br>38.41±2.00	0.9608±0.0081<br>38.80±2.19	**0.9615±0.0082<br>38.90±2.24**
		Sagittal	0.8176±0.0109<br>32.10±1.26	0.9524±0.0092<br>36.87±1.99	0.9550±0.0084<br>37.65±2.14	0.9588±0.0082<br>38.15±2.38	**0.9601±0.0082<br>38.27±2.43**
		Coronal	0.8151±0.0104<br>33.02±1.04	0.9533±0.0090<br>37.33±2.10	0.9560±0.0082<br>38.16±2.16	0.9599±0.0080<br>38.63±2.35	**0.9611±0.0081<br>38.75±2.40**
	$36EG$	Axial	0.8843±0.0104<br>34.66±0.83	0.9726±0.0068<br>40.25±2.32	0.9742±0.0063<br>41.08±2.42	0.9762±0.0059<br>41.31±2.44	**0.9766±0.0059<br>41.40±2.46**
		Sagittal	0.8853±0.0107<br>35.93±1.15	0.9711±0.0069<br>39.70±2.40	0.9728±0.0064<br>40.66±2.71	0.9749±0.0060<br>40.95±2.75	**0.9756±0.0060<br>41.06±2.78**
		Coronal	0.8840±0.0105<br>36.72±0.77	0.9718±0.0068<br>40.19±2.48	0.9734±0.0063<br>41.12±2.65	0.9756±0.0059<br>41.40±2.64	**0.9762±0.0059<br>41.51±2.67**
	$72EG$	Axial	0.9335±0.0088<br>37.98±0.80	0.9815±0.0039<br>42.45±1.32	0.9824±0.0034<br>43.00±1.64	0.9838±0.0031<br>43.34±1.71	**0.9841±0.0031<br>43.43±1.72**
		Sagittal	0.9330±0.0090<br>39.09±1.05	0.9804±0.0040<br>42.22±1.36	0.9813±0.0036<br>42.86±1.88	0.9829±0.0033<br>43.28±2.00	**0.9833±0.0032<br>43.39±2.02**
		Coronal	0.9331±0.0089<br>39.75±0.87	0.9810±0.0039<br>42.72±1.46	0.9819±0.0035<br>43.30±1.87	0.9834±0.0032<br>43.69±1.96	**0.9838±0.0031<br>43.80±1.98**
In-plane :5° Through-plane: 5°	$9EG$	Axial	0.7391±0.0164<br>27.90±1.28	0.9319±0.0135<br>35.59±1.91	0.9349±0.0118<br>36.03±1.99	0.9428±0.0100<br>36.65±1.94	**0.9442±0.0100<br>36.79±2.00**
		Sagittal	0.7448±0.0164<br>28.89±1.24	0.9277±0.0135<br>34.47±1.92	0.9296±0.0118<br>35.1±2.03	0.9390±0.0100<br>35.81±2.05	**0.9417±0.0100<br>35.97±2.11**
		Coronal	0.7393±0.0163<br>30.04±1.22	0.9285±0.0132<br>35.08±2.06	0.9308±0.0116<br>35.72±2.11	0.9402±0.0098<br>36.37±2.14	**0.9428±0.0098<br>36.53±2.20**
	$18EG$	Axial	0.8051±0.0126<br>30.49±1.24	0.9538±0.0091<br>37.31±1.84	0.9564±0.0081<br>37.96±1.94	0.9601±0.0078<br>38.31±2.05	**0.9609±0.0078<br>38.41±2.09**
		Sagittal	0.8072±0.0127<br>31.66±1.32	0.9506±0.0093<br>36.40±1.81	0.9529±0.0084<br>37.19±1.98	0.9574±0.0079<br>37.63±2.12	**0.9590±0.0079<br>37.75±2.17**
		Coronal	0.8042±0.0123<br>32.71±1.19	0.9514±0.0090<br>36.97±1.97	0.9539±0.0081<br>37.76±2.08	0.9584±0.0077<br>38.20±2.16	**0.9598±0.0078<br>38.32±2.21**
	$36EG$	Axial	0.8798±0.0101<br>34.27±0.90	0.9726±0.0064<br>40.03±1.92	0.9742±0.0065<br>40.68±2.24	0.9760±0.0068<br>41.03±2.53	**0.9764±0.0068<br>41.13±2.56**
		Sagittal	0.8801±0.0105<br>35.53±1.22	0.9708±0.0066<br>39.43±1.99	0.9721±0.0067<br>40.21±2.48	0.9744±0.0069<br>40.73±2.80	**0.9751±0.0069<br>40.85±2.83**
		Coronal	0.8786±0.0103<br>36.40±0.74	0.9713±0.0064<br>40.02±2.05	0.9728±0.0066<br>40.75±2.47	0.9750±0.0069<br>41.24±2.77	**0.9757±0.0069<br>41.36±2.80**
	$72EG$	Axial	0.9301±0.0086<br>37.56±0.80	0.9804±0.0039<br>41.54±1.43	0.9815±0.0035<br>42.14±1.77	0.9830±0.0032<br>42.47±1.73	**0.9834±0.0031<br>42.57±1.75**
		Sagittal	0.9291±0.0090<br>38.70±1.01	0.9789±0.0041<br>41.15±1.66	0.9799±0.0038<br>41.89±2.21	0.9818±0.0033<br>42.28±2.25	**0.9823±0.0033<br>42.38±2.28**
		Coronal	0.9290±0.0088<br>39.47±1.03	0.9795±0.0040<br>41.79±1.55	0.9805±0.0036<br>42.47±2.00	0.9823±0.0032<br>42.83±2.00	**0.9828±0.0032<br>42.94±2.03**
